# Combined acid hydrolysis and fermentation improves bioactivity of citrus flavonoids in vitro and in vivo

**DOI:** 10.1038/s42003-023-05424-7

**Published:** 2023-10-25

**Authors:** Alice König, Nadiia Sadova, Marion Dornmayr, Bettina Schwarzinger, Cathrina Neuhauser, Verena Stadlbauer, Melanie Wallner, Jakob Woischitzschläger, Andreas Müller, Rolf Tona, Daniel Kofel, Julian Weghuber

**Affiliations:** 1 Center of Excellence Food Technology and Nutrition, University of Applied Sciences Upper Austria, Stelzhamerstraße 23, Wels, 4600 Austria; 2 FFoQSI GmbH-Austrian Competence Centre for Feed and Food Quality, Safety and Innovation, Technopark 1D, Tulln, 3430 Austria; 3 TriPlant AG, Industriestrasse 17, Buetzberg, 4922 Switzerland

**Keywords:** Microbiology techniques, Hydrolases, Assay systems

## Abstract

Many bioactive plant compounds, known as phytochemicals, have the potential to improve health. Unfortunately, the bioavailability and bioactivity of phytochemicals such as polyphenolic flavonoids are reduced due to conjugation with sugar moieties. Here, we combine acid hydrolysis and tailored fermentation by lactic acid bacteria (*Lactiplantibacillus plantarum*) to convert the biologically less active flavonoid glycosides hesperidin and naringin into the more active aglycones hesperetin and naringenin. Using a comprehensive approach, we identify the most effective hydrolysis and fermentation conditions to increase the concentration of the aglycones in citrus extracts. The higher cellular transport and bioactivity of the biotransformed citrus extract are also demonstrated in vitro and in vivo. Superior antioxidant, anti-inflammatory and cell migration activities in vitro, as well as intestinal barrier protecting and antioxidant activities in *Drosophila melanogaster* are identified. In conclusion, the presented biotransformation approach improves the bioactivity of flavonoids, clearly traced back to the increase in aglycone content.

## Abstract (web summary)

Combined acid hydrolysis and fermentation by *Lactiplantibacillus plantarum* of citrus extract promoted flavonoids’ deglycosylation and improved cellular transport and bioactivity of the biotransformed extract in vitro and in *Drosophila melanogaster*.

## Introduction

The health-promoting effects of secondary plant compounds, known as phytochemicals, have been a major focus of plant research in recent years^1,2^. For instance, citrus flavonoids, polyphenolic phytochemicals found in plants of the *Rutaceae* family such as sweet oranges (*Citrus sinensis*), bitter oranges (*Citrus aurantium*) or grapefruits (*Citrus paradisi*) have been of special interest^3–5^ due to their anti-inflammatory^5–7^ and antioxidant^4,5^ properties. One subclass of flavonoids are flavanones with naringin and hesperidin as the most abundant representatives^8^. These flavanones naturally occur in their glycosidic form, in which the respective aglycones naringenin and hesperetin are bound to a sugar moiety that is either a neohesperidose (2-*O*-L-rhamnosyl-D-glucose) or a rutinose (6-*O*-L-rhamnosyl-D-glucose)^5,8,9^. These sugar moieties prevent an efficient absorption in the small intestine^8,9^ and therefore reduce the bioavailability and bioactivity of flavanones^5,10,11^. Intact flavonoids enter the colon, where they undergo extensive metabolism by the intestinal microbiota^8^.

**Fig. 1:**
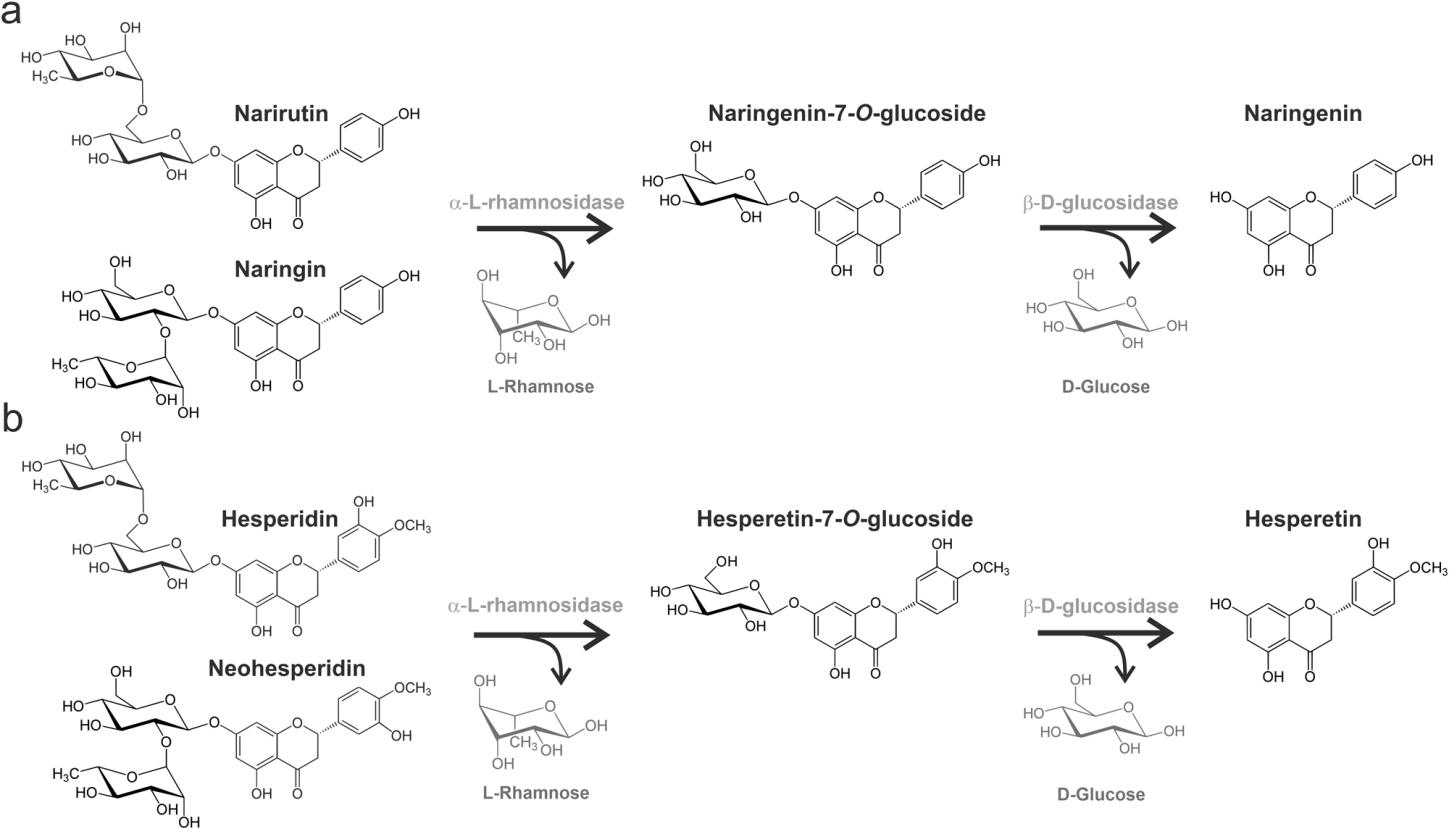
Degradation of flavanones by α-L-rhamnosidase and β-D-glucosidase. **a** Narirutin (naringenin-7-*O*-rutinoside) or naringin (naringenin-7-*O*-neohesperidoside) are converted to naringenin-7-*O*-glucoside by α-L-rhamnosidase. Next, β-D-glucosidase catalyzes the conversion of naringenin-7-*O*-glucoside to naringenin. **b** Hesperidin (hesperetin-7-*O*-rutinoside) or neohesperidin (hesperetin-7-*O*-neohesperidoside) are hydrolyzed to hesperetin-7-*O*-glucoside by α-L-rhamnosidase. Hesperetin-7-*O*-glucoside is converted to hesperetin by β-D-glucosidase.

One strategy to improve bioavailability and bioactivity of flavonoids is to increase aglycone levels by deglycosylation^12,13^. A randomized, double-blind crossover trial with human subjects could show that enzymatic removal of the sugar moiety from hesperidin in orange juice changes the site of absorption from colon to the small intestine, thereby improving flavanone bioavailability^12^. The classical approach for conversion of citrus flavanones requires the hydrolases α-L-rhamnosidase (EC 3.2.1.40) and β-D-glucosidase (EC 3.2.1.21)^9^. As shown in Fig. 1, α-L-rhamnosidase first cleaves terminal L-rhamnose from narirutin and naringin (1a) or hesperidin and neohesperidin (1b) and converts them to naringenin-7-*O*-glucoside or hesperetin-7-*O*-glucoside, respectively. Next, β-D-glucosidase splits off D-glucose and releases the aglycones naringenin and hesperetin. A sustainable and cost-efficient alternative to enzymes is fermentation, in which molecules are degraded by the enzymatic activities of microorganisms. Lactic acid bacteria (LAB), especially *Lactobacillus* species, are of particular interest here, as they catabolize flavonoids in the human colon^8^. Many *Lactobacillus* species, such as *Lactiplantibacillus plantarum* (*L. plantarum*) are generally recognized as safe by the United States Food and Drug Administration^14^ and included to the list of biological agents with qualified presumption of safety by the European Food Safety Authorities^15^, justifying their use in food and feed. Previous studies have demonstrated that various *Lactobacillus* species can cleave flavanones^9,16,17^, however, the reported conversion took 10 days and the aglycone yield was moderate^9^.

In the present study, we aimed to accelerate the conversion of biologically less active flavonoid glycosides into more active aglycones using a combination of acid hydrolysis and LAB fermentation of citrus extracts. Our purpose was not to develop a highly specific hydrolysis method, but to establish a biotransformation method that can be used in the food and feed industry as an alternative to commercially available enzymes. In addition, we wanted to determine whether the biotransformation of flavonoids changes the bioactivity of the citrus extracts. For this purpose, we implemented suitable in vitro and in vivo models to study flavonoid transport and uptake, antioxidant and anti-inflammatory properties, as well as intestinal barrier integrity. A requirement for bioactivity is that the substances are available to the cells. Thus, we first tested the uptake and transport of flavonoids from our extracts in the human intestinal epithelial cell line Caco-2. Differentiated Caco-2 cells express tissue-typical cell membrane and transport proteins and have been established as in vitro model to study drug absorption^18,19^. Since oxidative stress is a major cause of barrier disruption and inflammation in the small intestine^20^, experiments on oxidative stress and cell migration were performed in the intestinal cell lines, Caco-2 and IPEC-J2. The porcine IPEC-J2 intestinal epithelial cell line is a valuable tool to study the effects of phytochemicals on intracellular oxidative stress and wound healing^20,21^. Cell migration assays are simplified in vitro models of wound healing and require cells with high migration rates, such as IPEC-J2, to differentiate between cell migration and cell division^22^. The anti-inflammatory activities of flavanones were determined in human monocytic THP-1 cells, a suitable model to study inflammatory immune responses because differentiated THP-1 cells behave similarly to native monocyte-derived macrophages^22,23^. To verify whether deglycosylation of flavanones in biotransformed citrus extracts protects the intestinal barrier in vivo, we used the fruit fly *Drosophila melanogaster (D. melanogaster)* model. The midgut of *D. melanogaster* is well comparable with the mammalian small intestine^24–27^ making the fruit fly a valuable model organism for studying the effects of bioactive compounds on the intestinal barrier. We also investigated the antioxidant properties of glycosylated flavanones and their aglycones in the fruit fly, as *D. melanogaster* is a well-established model in oxidative stress research^28–30^.

Taken together, we demonstrated an efficient method of flavanone biotransformation via a combination of acid hydrolysis and 24 h-fermentation and provided a comprehensive approach to study the bioactivity of these biotransformed flavanones. Superior antioxidant and anti-inflammatory activities were observed for biotransformed citrus extracts with increased hesperetin and naringenin content.

## Results

### Combination of acid hydrolysis and fermentation increases aglycone content in citrus extracts

First, we confirmed the degradation process described in Fig. 1 by incubating an aqueous extract (AQE) made of dried citrus fruits containing all eight flavanones of interest (naringin, neohesperidin, hesperidin, narirutin, naringenin-7-*O*-glucoside, hesperetin-7-*O*-glucoside, naringenin and hesperetin) with the enzymes α-rhamnosidase or β-glucosidase. Our results indicated that α-L-rhamnosidase from prokaryotic source was able to hydrolyze the rutinosides narirutin and hesperidin, in which L-rhamnose is α-1,6-linked to β-D-glucoside, but did not cleave the neohesperidosides naringin and neohesperidin, in which L-rhamnose is α-1,2-linked to β-D-glucoside (Supplementary Figure 1a). In contrast, β-D-glucosidase increased naringenin and hesperetin by degradation of naringenin-7-*O*-glucoside and hesperetin-7-*O*-glucoside, respectively (Supplementary Figure 1b).

Next, we screened four different LAB strains, namely *L. plantarum* DSM 20205, *Lacticaseibacillus rhamnosus* NCTC 10302 (*L. rhamnosus*), *Levilactobacillus brevis* DSM 6235 (*L. brevis*) and *Lacticaseibacillus paracasei* DSM 20312 (*L. paracasei*), to determine whether they produce the enzymes required for increasing the aglycone levels in AQE. The specific LAB strains were selected based on literature research^16,17,31^ and their safety for use in food and feed. Bacterial growth was not inhibited by the concentration of citrus extract used (5% of citrus extract; Supplementary Figure 2). Although flavonoids have a low water solubility, water was used as solvent for citrus extract preparation because other organic solvents such as ethanol would have greatly inhibited bacterial growth during fermentation. After fermentation, the citrus extracts were centrifuged to remove the bacteria from the extract. Thus, the stated concentrations refer to flavonoids dissolved in the supernatant.

All tested bacteria were able to convert naringenin-7-*O*-glucoside and hesperetin-7-*O*-glucoside into the aglycones naringenin and hesperetin, respectively. The highest aglycone increase with 168 µM of naringenin and 105 µM of hesperetin was observed after 24 h of fermentation with *L. rhamnosus*. However, the flavanones naringin, neohesperidin, narirutin and hesperidin were hardly metabolized during incubation with bacteria for 144 h (6 d) (Supplementary Figure 3a). These findings led us to the assumption that all strains have β-glucosidase activity but no noticeable α-rhamnosidase activity. Addition of 2% of glucose to AQE improved bacterial growth (Supplementary Figure 3b) but reduced the β-glucosidase activity of the bacteria resulting in significantly lower aglycone levels (Supplementary Figure 3c). Thus, medium without glucose was used for fermentation of extracts.

**Fig. 2:**
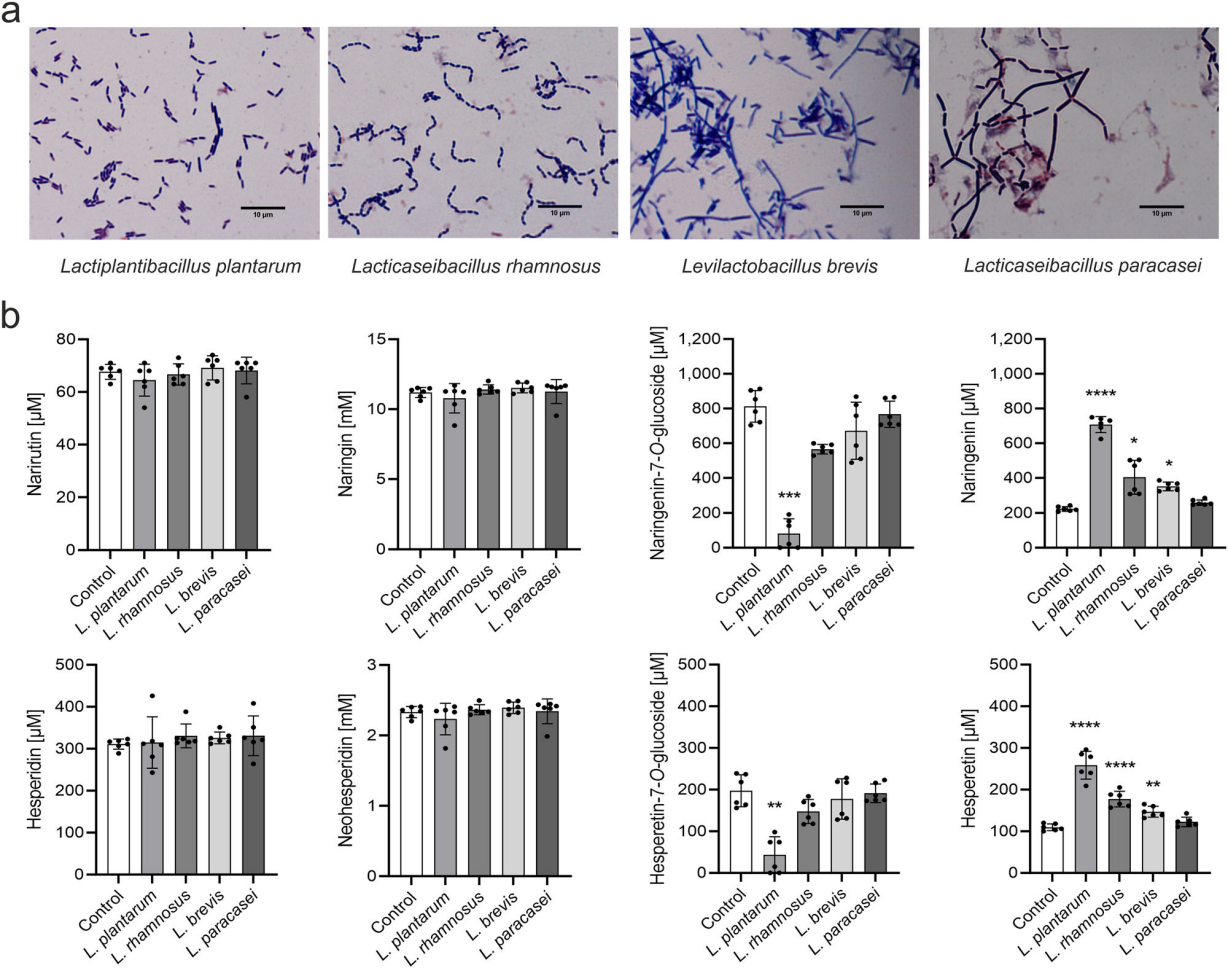
Enzymatic activity of various LAB strains for the formation of aglycones from 7-*O*-glucosides present in citric acid hydrolyzed citrus extract. **a** Representative images of *L. plantarum, L. rhamnosus, L. brevis* and *L. paracasei* showing gram-positive rods. Scale bar: 10 µm. **b** Concentrations of flavanones in citric acid hydrolyzed extract (CAE) after 24 h incubation with each strain at 37 °C. Control refers to the sample without bacteria. Data are mean ± SD of *n* = 6 samples/strain. Differences to control are analyzed by Kruskal-Wallis’s test with Dunn’s multiple comparisons test, with exception of data set for hesperetin which is analyzed by ordinary one-way ANOVA with Dunnett’s multiple comparison test.

**Fig. 3:**
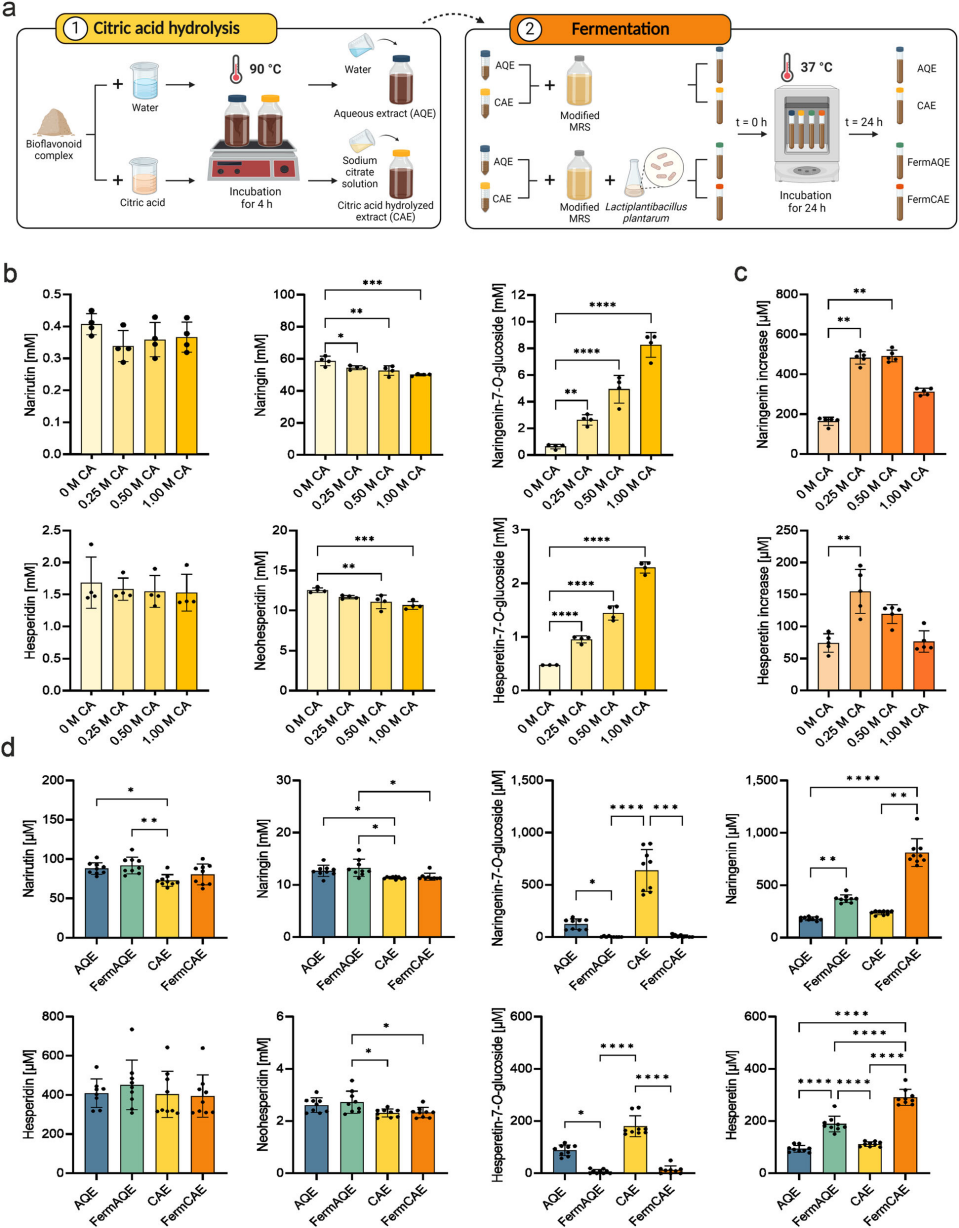
Citric acid-based hydrolysis and fermentation affect flavanone profiles in citrus extracts. **a** Schematic diagram of extract preparation. For citric acid hydrolyzed extract (CAE), bioflavonoid complex was treated with citric acid solution at 90 °C for 4 h. Then, sodium citrate solution was added to reach a final concentration of 10% (w/w). Aqueous extract (AQE) was prepared with water instead of citric acid. For fermentation, AQE and CAE were incubated with *L. plantarum* at 37 °C for 24 h (referred to as FermAQE and FermCAE). Control extracts were diluted in modified MRS and incubated without bacteria (referred to as AQE and CAE). **b** Influence of hydrolysis with increasing citric acid (CA) concentrations (0.25 M CA, 0.50 M CA, or 1.00 M CA) on flavanone concentrations in citrus extracts before incubation with bacteria. Data are mean ± SD of *n* = 4 samples/treatment. Differences to 0 M CA are analyzed by ordinary one-way ANOVAs with Dunnett’s multiple comparison test, with exception of data set for hesperidin which is analyzed with Kruskal’s test with Dunn’s multiple comparisons test. **c** Increase in aglycones after 24 h-incubation with *L. plantarum*. For calculation of the aglycone increase, the initial aglycone concentration (at 0 h) was subtracted from the total concentration at 24 h. Data are mean ± SD of *n* = 5 samples/treatment. Differences to 0 M CA are analyzed by Kruskal’s test with Dunn’s multiple comparisons test. **d** Flavonoid concentrations in the final extracts prepared with water (AQE and FermAQE) or 0.25 M CA (CAE and FermCAE). Data are mean ± SD of *n* = 9 samples/extract. Differences between treatments are analyzed by ordinary one-way ANOVAs with Tukey’s multiple comparison test (narirutin, neohesperidin and hesperetin) or Kruskal’s test with Dunn’s multiple comparisons test (naringin, naringenin-7-*O*-glucoside, naringenin, hesperidin and hesperetin-7-*O*-glucoside).

Next, we enhanced the efficiency of glycolytic cleavage by adding acid hydrolysis. First, the L-rhamnose group was split off from naringin, neohesperidin, hesperidin and narirutin using citric acid, resulting in citric acid hydrolyzed extract (CAE). In a next step, the resulting 7-*O*-glucosides, namely naringenin-7-*O*-glucoside and hesperetin-7-*O*-glucosides, were converted to aglycones by the bacterial β-glucosidase activity. A fermentation period of 24 h was chosen because a longer incubation time did not show any improvement in our previous experiments. As presented in Fig. 2b, fermentation of CAE with bacteria resulted in significantly higher aglycone levels compared to the control treatment incubated without bacteria. The maximum increases in naringenin (463 µM) and hesperetin (144 µM) above the initial concentrations were achieved by fermenting CAE with *L. plantarum*. Thus, this strain was used for preparation of the final extracts (Fig. 3a). As shown in Supplementary Figure 4, co-cultivation of *L. plantarum* with different LAB strains did not improve the aglycone yield significantly.

Next, we performed acid hydrolysis with different citric acid concentrations to optimize the deglycosylation process. We first aimed to increase the content of 7-*O*-glucosides by acid hydrolysis so that they could be converted to aglycones by bacterial β-glucosidase activity. As shown in Fig. 3b, citric acid hydrolysis significantly enhanced the levels of 7-*O*-glucosides in a concentration-dependent manner. The higher the acid concentration, the more efficient the hydrolysis of naringin to naringenin-7-*O*-glucoside as well as neohesperidin to hesperetin-7-*O*-glucoside was. Subsequent fermentation by *L. plantarum* significantly reduced these 7-*O*-glucosides to aglycones (Fig. 3c). The increase of naringenin and hesperetin was significantly higher in fermented extract that was previously treated with 0.25 M of citric acid compared to fermented aqueous extract (FermAQE; 0 M citric acid). Contrary to expectations, higher initial 7-*O*-glucoside levels due to higher citric acid concentrations (0.50 M or 1.00 M) did not improve the final aglycone content, indicating limited β-D-glucosidase activity. Thus, we used the lowest citric acid concentration (0.25 M) to prepare the final CAE and fermented citric acid hydrolyzed extracts (FermCAE). Flavanone profiles of the four final extracts (AQE, FermAQE, CAE and FermCAE) that were used for in vitro and in vivo experiments are provided in Fig. 3d and representative chromatograms in Supplementary Figure 5. Citric acid hydrolysis proved to be an effective way to increase the overall yield of 7-*O*-glucosides since CAE contained the highest naringenin-7-*O*-glucoside (638 ± 201 µM) and hesperetin-7-*O*-glucoside (180 ± 40 µM) levels. Nevertheless, the maximum content of aglycones was achieved after fermentation in FermCAE (812 ± 132 µM of naringenin and 291 ± 30 µM of hesperetin), which was 4.5-fold (naringenin) and 3.1-fold (hesperetin) higher than the aglycone content in AQE.

As mentioned above, only dissolved flavonoids in the supernatant after centrifugation were considered for our final extracts. However, the entire fermentation product also includes precipitated flavonoids in the residue. We could show that the percentage of flavonoids in the pellet was low, with a maximum of 2.20% of the total flavonoid content (FermCAE). Of these, aglycones accounted for the largest proportion with 0.63% (FermCAE). Supplementary Figure 6 summarizes the proportions of flavonoids in supernatant and pellet for all extracts.

Taken together, the combination of citric acid hydrolysis and fermentation with *L. plantarum* was proven to be the most effective method to transform glycosides into aglycones in the citrus extract.

### Intestinal epithelial cells take up and transport more bioactive flavonoids from biotransformed citrus extracts

**Fig. 4:**
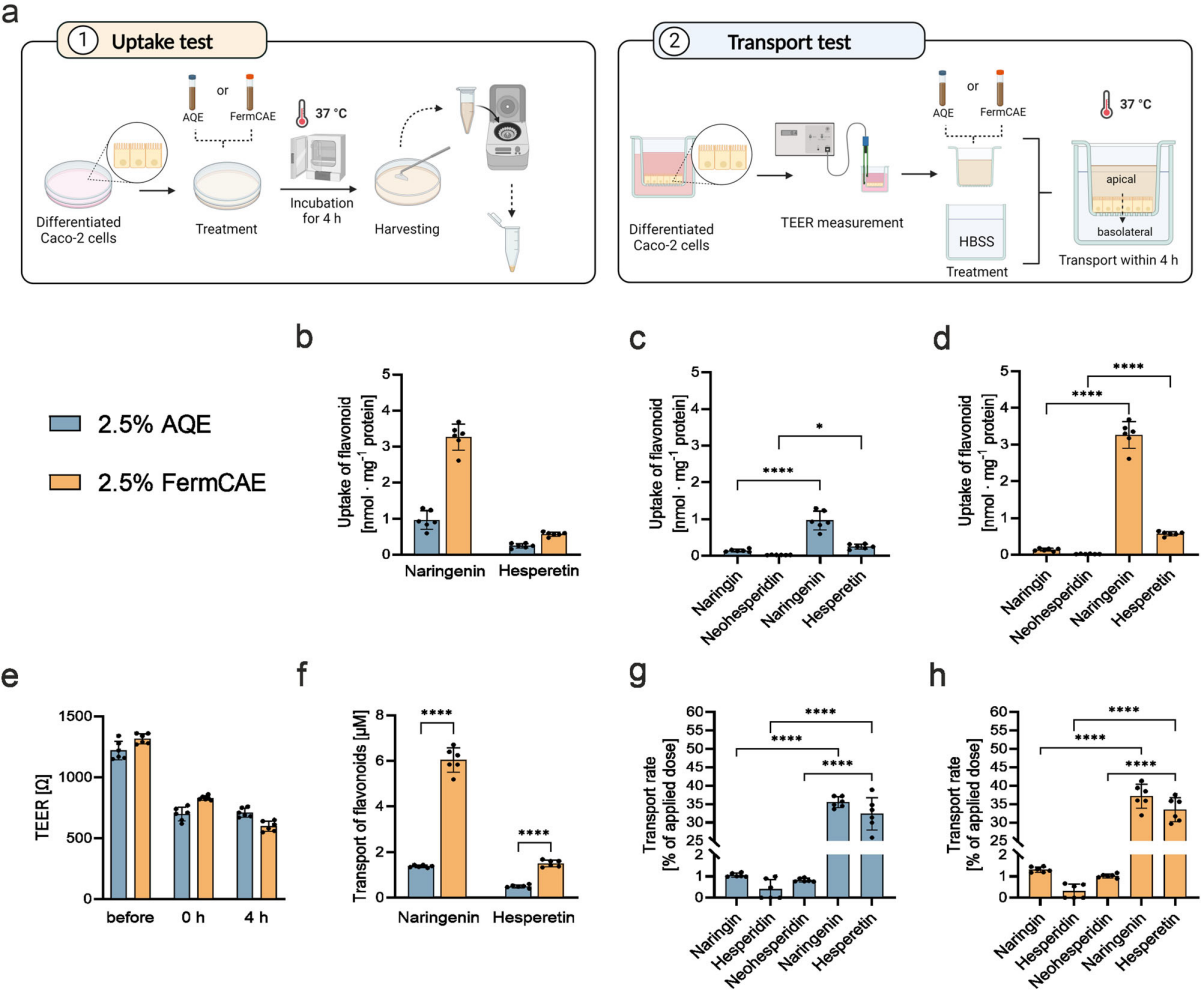
Transport and uptake of flavonoids from AQE or FermCAE in Caco-2 cells. **a** Schematic overview of uptake test (**1**) or transport test (**2**) in differentiated Caco-2 cells. **b** Comparison of aglycone uptake in Caco-2 cells treated with AQE or FermCAE for 4 h, normalized to cell protein content. Uptake of detectable flavonoids from (**c**) aqueous citrus extract (AQE) and (**d**) fermented citric acid hydrolyzed citrus extract (FermCAE) in Caco-2 cells, normalized to cell protein content. **e** TEER values of Caco-2 monolayers before and during transport studies. **f** Comparison of absolute aglycone concentrations detected on the basolateral side after treatment with AQE or FermCAE on the apical side for 4 h. Transport rate of detectable flavonoids from AQE (**g**) and FermCAE (**h**) across a Caco-2 monolayer. Data are mean ± SD of *n* = 6 samples/treatment. Differences are analyzed by ordinary one-way ANOVAs with Šídák’s multiple comparison test.

To demonstrate, whether the amount of aglycones present in biotransformed citrus extracts is bioavailable to intestinal epithelial cells, we investigated the cellular uptake and transport of flavanones from AQE and FermCAE in differentiated Caco-2 cells. For uptake tests, the cells were incubated with AQE or FermCAE (2.5% in Hank’s balanced salt solution; HBSS) for 4 h (Fig. 4a, 1). First, we could prove that cellular uptake of the aglycones naringenin and hesperetin was significantly better than uptake of glycosylated flavonoids in both AQE and FermCAE (Fig. 4c, d). Moreover, when cells received higher concentrations of hesperetin and naringenin in form of FermCAE (Fig. 4b), they absorbed significantly higher absolute amounts of these aglycones (3.26 and 0.57 nmol · mg^-1^ protein for naringenin and hesperetin) compared to AQE (0.96 and 0.25 nmol · mg^−1^ protein, respectively).

For transport tests, Caco-2 monolayers were incubated with AQE or FermCAE (2.5% in HBSS) for 4 h and the transport of flavanones from the apical to the basolateral compartment was determined (Fig. 4a, 2). The monolayer integrity was controlled by transepithelial electrical resistance (TEER) measurements. As shown in Fig. 4e, TEER values remained above 500 Ω throughout the experiment indicating intact monolayers. We could confirm that the aglycones have higher transport rates (above 32%) than their respective glycosylated forms (below 2%). More importantly, we showed that this remains true when flavonoids were applied as a mixture in form of the citrus extracts AQE and FermCAE (Fig. 4g, h). When epithelial cells received higher concentrations of flavonoid aglycones in FermCAE, they could transfer significantly higher amounts of hesperetin and naringenin compared to AQE (*p* < 0.0001; Fig. 4f). Neither narirutin nor the 7-*O*-glucosides could be detected in the basolateral compartment after 4 h.

Our findings showed that citric acid hydrolysis and fermentation of citrus extract did not change the dynamics of cellular uptake or hinder cellular transport. On the contrary, we demonstrated that epithelial cells could take up and transport higher absolute amounts of flavonoids without reaching saturation within the concentrations in FermCAE. Taken together, higher flavonoid contents, provided by biotransformed citrus extract, resulted in a significantly higher uptake and transport creating conditions for superior bioactivity of FermCAE.

### Biotransformed citrus extracts reduce oxidative stress and improve cell migration of intestinal cells under challenging conditions

**Fig. 5:**
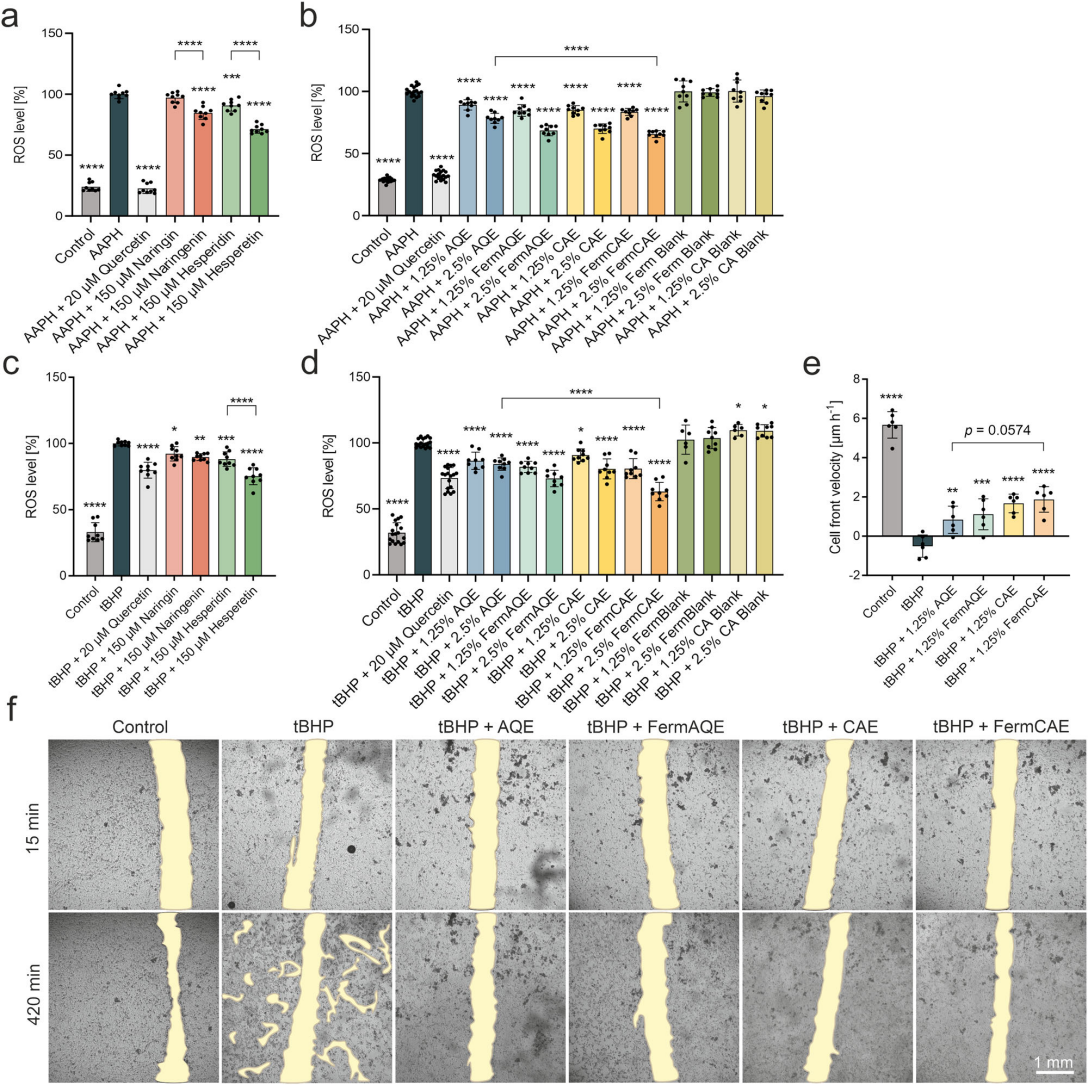
Impact of citrus extracts of different biotransformation level on ROS production and cell migration of intestinal epithelial cells under challenging conditions. Percentage of intracellular reactive oxygen species (ROS) accumulation in human Caco-2 cells after treatment with pure flavanones (**a**), or citrus extracts (**b**) and subsequent stress induction by 2,2’-azobis (2-amidinopropane) dihydrochloride (AAPH), normalized to stressor treatment. AQE stands for aqueous citrus extract, CAE for citric acid hydrolyzed citrus extract, FermAQE for fermented aqueous citrus extract, FermCAE for fermented citric acid hydrolyzed citrus extract. Percentage of intracellular ROS accumulation in swine epithelial IPEC-J2 cells after treatment with pure flavanones (**c**) or citrus extracts (**d**) and subsequent stress induction by tert-butylhydroperoxide (tBHP), normalized to stressor treatment. Control refers to untreated cells; quercetin was used as antioxidant positive control. Data are mean ± SD of *n* = 9 samples/treatment. Differences are analyzed by ordinary one-way ANOVAs with Šídák’s multiple comparison test. **e** Cell front velocity [µm · h^−1^] of IPEC-J2 cells pre-treated with citrus extracts for 6 h and stressed with tBHP after scratching. Data are mean ± SD of *n* = 6 samples/treatment. Differences are analyzed by ordinary one-way ANOVAs with Šídák’s multiple comparison test. **f** Representative brightfield images of the scratch-wounded area at start (15 min after scratching) and end point (420 min). The wounded area is highlighted in yellow ocher.

The influence of individual flavanones (naringin, naringenin, hesperidin and hesperetin) and the four citrus extracts of different biotransformation level (AQE, FermAQE, CAE and FermCAE) on cell viability and intracellular reactive oxygen species (ROS) formation was investigated in Caco-2 and IPEC-J2 cells. For ROS measurements, Caco-2 and IPEC-J2 cells were first treated with the phytochemicals and then with suitable stressors, respectively. The applied concentrations of pure flavanones (150 µM) or extracts (1.25% and 2.5%) and subsequent treatment with the respective stressor did not affect cell viability (Supplementary Table 1). We compared antioxidant effects of glycosylated flavanones (naringin and hesperidin) with their respective aglycones (naringenin and hesperetin) when applied at the same concentration (flavonoid stocks prepared in dimethyl sulfoxide, DMSO). Both aglycones, naringenin and hesperetin, exhibited a significantly stronger reduction of ROS formation in stressed Caco-2 cells than their glycosylated forms naringin and hesperidin (Fig. 5a). Along with this, the biotransformed extract with the highest aglycone content, FermCAE at a concentration of 2.5%, reduced ROS levels in stressed Caco-2 cells by 34%, being significantly different to AQE (*p* < 0.0001) at the same concentration (Fig. 5b). Similar findings were obtained for stressed IPEC-J2 cells. Here, the difference between hesperidin (ROS reduction by 12%) and its aglycone hesperetin (ROS reduction by 25%) was significant with *p* < 0.0001 (Fig. 5c). In addition, FermCAE at a concentration of 2.5% had the best antioxidant effect, reducing ROS by 37% (Fig. 5d). This was significantly different from the non-biotransformed extract AQE with ROS reduction by 16% at a concentration of 2.5% (*p* < 0.0001). Samples containing only bacteria (FermBlank) or citric acid solution (CA Blank) without flavanones did not reduce ROS formation in stressed intestinal epithelial cells. This finding suggests that the superior effect of FermCAE over other citrus extracts was due to its higher aglycone content.

Since high ROS levels are associated with impaired wound healing^32^, we next investigated whether the extracts could also improve cell migration and thereby potentially wound healing capacity of IPEC-J2 cells, using a scratch assay. For this purpose, cells were pre-treated with 1.25% of citrus extracts for 6 h. After scratching, cells were incubated with the stressor in presence of the citrus extracts and cell migration was recorded by automated time lapse microscopy for 7 h (Supplementary Movie). To compare cell migration between treatments, the cell front velocity, defined as the speed at which cells move toward each other, was calculated.

As shown in Fig. 5e, f, stressor treatment strongly impaired cell migration of IPEC-J2 cells. In contrast, all citrus extracts restored cell migration ability, as evident by a significant rise in cell front velocities compared to stressor treatment. The best effect was observed with FermCAE, which increased cell front velocity by 2.38 µm · h^−1^ (*p* < 0.0001). In comparison, the effect of AQE was lower, showing an increase in cell front velocity by 1.35 µm · h^−1^ with *p* = 0.0069, but not significantly different from FermCAE.

To summarize, these data indicate that the increase of naringenin and hesperetin in biotransformed citrus extracts leads to an enhanced reduction of oxidative stress and improved cell migration compared to the respective non-biotransformed extracts.

### Biotransformed citrus extracts attenuate LPS-induced inflammation in macrophages

**Fig. 6:**
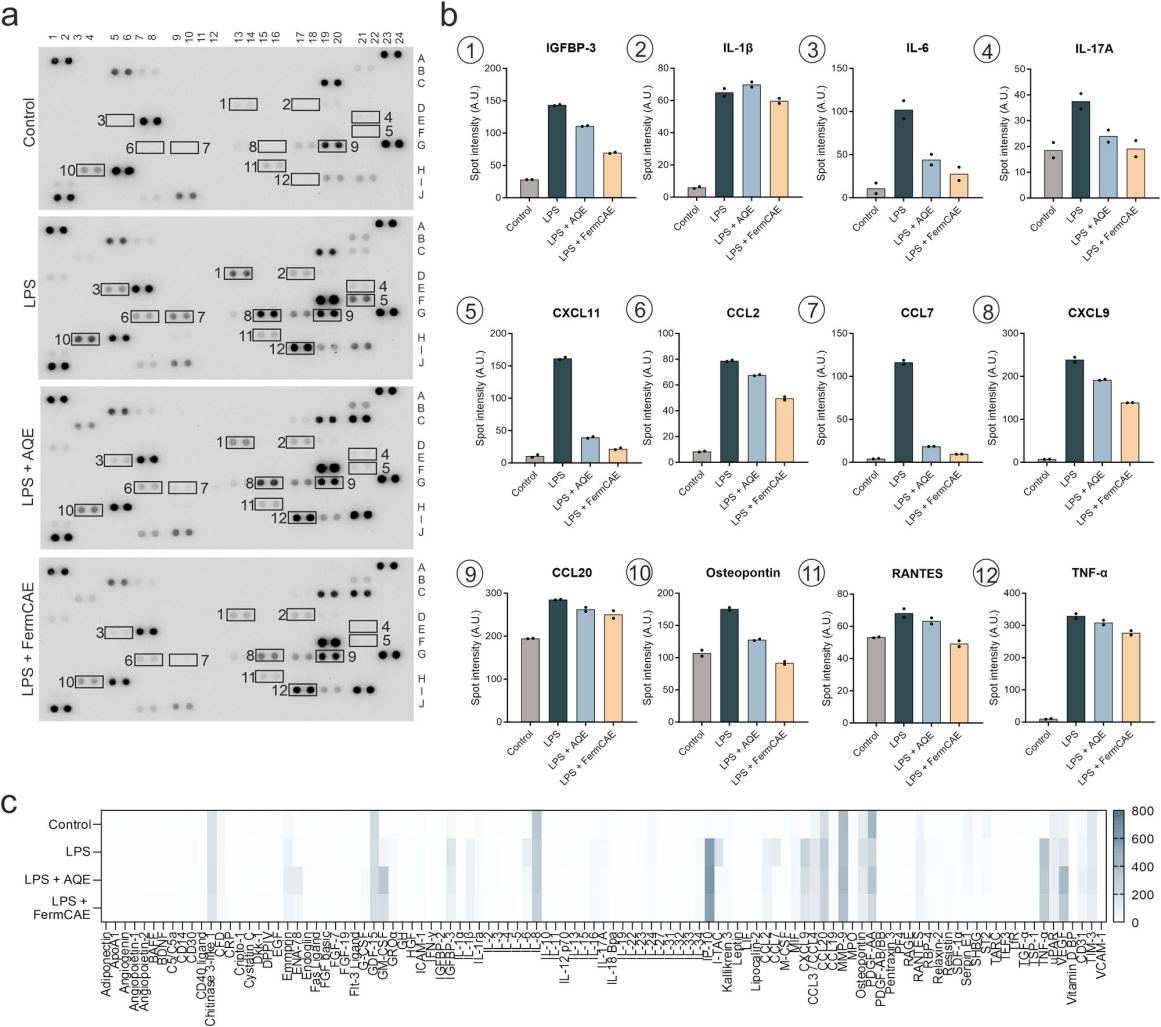
Change in cytokine expression profiles in LPS-stimulated THP-1 macrophages treated with citrus extracts of different biotransformation level. **a** Representative images of cytokine arrays incubated with supernatants of differentiated THP-1 macrophages treated with aqueous citrus extract (AQE) or fermented citric acid hydrolyzed citrus extract (FermCAE) under lipopolysaccharide (LPS) challenge. Cytokines expressed are presented as duplicate spots. Important cytokines are outlined and marked with numbers. The corresponding spot intensities (stated as arbitrary units, A.U.) of these analytes are presented in **b**. Data of technical duplicates are shown. **c** Heat map of 105 cytokines with mean spot intensities in a color range from white (low level) to blue (high level).

To study the influence of investigated extracts on inflammatory processes, we first performed a semi-quantitative cytokine array analysis. Therefore, differentiated THP-1 macrophages were challenged with lipopolysaccharides (LPS) for 24 h to trigger an inflammatory response and simultaneously treated with 1.25% FermCAE or AQE. The concentrations were selected based on cytotoxicity testing (Supplementary Figure 7). Membranes pre-loaded with antibodies specific for 105 human cytokines were incubated with the supernatants of treated THP-1 cells. Captured analytes were then visualized as spots (Fig. 6a). Spot intensities of important cytokines that were upregulated upon LPS stimulation compared to control are presented in Fig. 6b, the overall expression profiles are summarized in a heat map in Fig. 6c. Both extracts, AQE and FermCAE, reduced the concentrations of insulin-like growth factor-binding protein 3 (IGFBP-3), interleukin 6 (IL-6), interleukin 17A (IL-17A), C-X-C motif chemokine ligand 11 (CXCL11), CC-chemokine ligand 2 (CCL2), CC-chemokine ligand 7 (CCL7), C-X-C motif chemokine ligand 9 (CXCL9), CC-chemokine ligand 20 (CCL20), osteopontin, regulated upon activation, normal T-cell expressed and presumably secreted (RANTES) and tumor necrosis factor α (TNF-α). The previously most promising extract FermCAE caused stronger reduction of these cytokines than the non-biotransformed extract AQE. Three cytokines, epithelial neutrophil-activating protein 78 (ENA-78 or CXCL5), granulocyte macrophage-colony stimulating factor (GM-CSF) and vascular endothelial-derived growth factor (VEGF), were upregulated by treatment with both extracts but not with LPS alone. Additional data of cytokine array analysis with the aglycone naringenin are provided in Supplementary Figure 8, showing a similar cytokine profile to the one with the extracts.

**Fig. 7:**
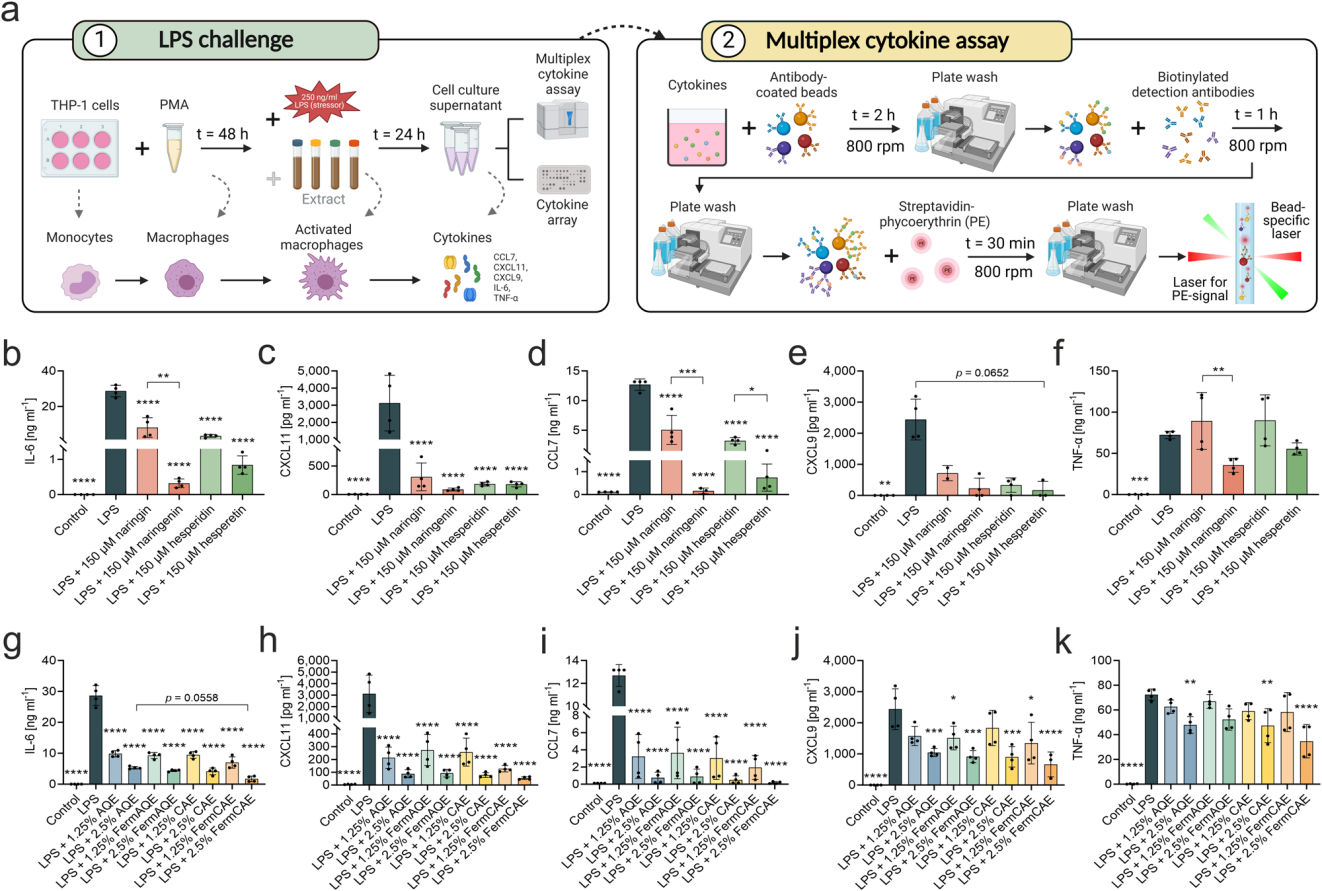
Biotransformed citrus extracts reduce pro-inflammatory cytokine levels in LPS-stimulated THP-1 macrophages. **a** Schematic graph of lipopolysaccharide (LPS) challenge of THP-1 cells (**1**) with subsequent multiplex bead-based cytokine immunoassay (**2**). AQE stands for aqueous citrus extract, CAE for citric acid hydrolyzed citrus extract, FermAQE for fermented aqueous citrus extract, FermCAE for fermented citric acid hydrolyzed citrus extract. Effect of pure flavonoids (**b**−**f**) and citrus extracts (**g**−**k**) on expression of selected pro-inflammatory cytokines by differentiated THP-1 macrophages. Data are mean ± SD of *n* = 4 samples/treatment, measured in technical duplicates. Differences between treatments are analyzed by ordinary one-way ANOVAs with Šídák’s multiple comparison test with exception of CXCL9 in pure flavonoid treatments (**e**), which is analyzed with Kruskal-Wallis’s test with Dunn’s multiple comparisons test due to not normally distributed data.

Based on our findings, we selected five cytokines for further investigation via bead-based multiplex immunoassay (Fig. 7a), namely IL-6, CXCL11, CCL7, CXCL9 and TNF-α. As expected, LPS significantly upregulated the secretion of these cytokines compared to unstimulated control. Concurrent treatment with pure flavanones significantly (*p* < 0.0001) reduced IL-6 (Fig. 7b), CXCL11 (Fig. 7c) and CCL7 (Fig. 7d) and showed a slight decrease in CXCL9 (Fig. 7e) and TNF-α (Fig. 7f). Distinctively, the anti-inflammatory effects of the aglycones naringenin and hesperetin were more pronounced than of their glycosylated forms. Significant differences between naringin and its aglycone naringenin were found for IL-6 (*p* = 0.0042), CCL7 (*p* = 0.0003) and TNF-α (*p* = 0.0076), while the aglycone hesperetin showed a significantly stronger reduction of CCL7 than hesperidin (*p* = 0.0497). Consistent with these data, flavonoid-rich extracts also strongly decreased IL-6, CXCL11, CCL7, CXCL9 and TNF-α (Fig. 7g–k) in a concentration-dependent manner. Furthermore, the extract with the highest aglycone content, FermCAE, reduced pro-inflammatory cytokines more efficiently than the other extracts.

In conclusion, the results of our in vitro experiments revealed that the aglycones naringenin and hesperetin have a higher bioactivity than respective glycosylated flavonoids found in citrus extracts. Accordingly, the increase of the aglycone concentration in citrus extracts improved their anti-inflammatory properties.

### Biotransformed citrus extracts reduce intestinal barrier damage and mortality in female *D. melanogaster*

**Fig. 8:**
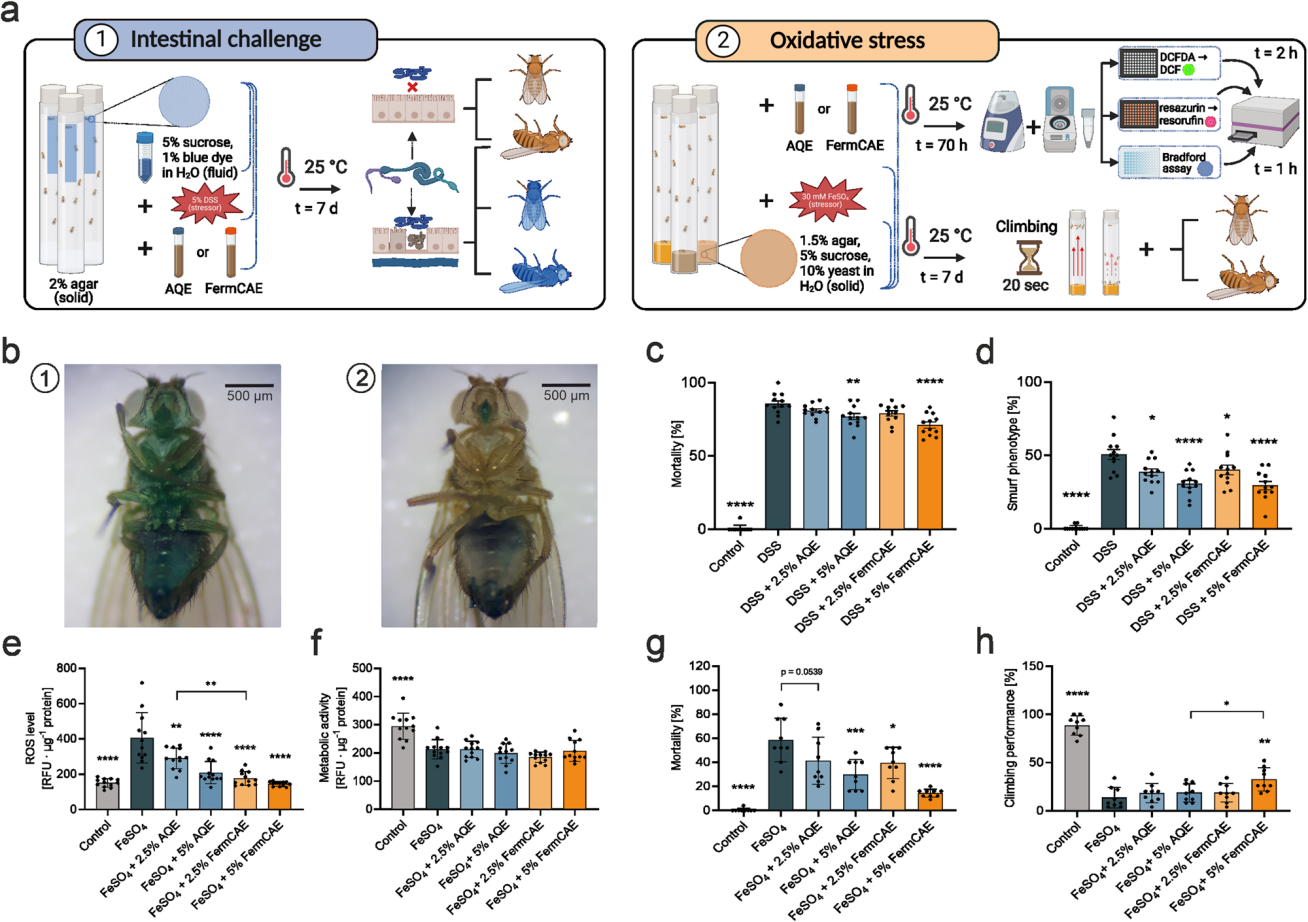
Biotransformed citrus extracts improve intestinal barrier integrity and reduce oxidative stress in female w^1118^*D. melanogaster* under challenge conditions. **a** Schematic overview of in vivo challenges: (**1**) dextran sulphate sodium (DSS) challenge to reduce intestinal barrier integrity; (**2**) ferrous iron induced oxidative stress in female *w*^*1118*^ *D. melanogaster*. AQE stands for aqueous citrus extract, FermCAE stands for fermented citric acid hydrolyzed citrus extract, DCF(DA) stands for 2’,7’-dichlorodihydrofluorescein (diacetate). **b** *D. melanogaster* fed with blue dye (**1**) with DSS-challenged intestinal barrier (Smurf phenotype) and (**2**) in control group. Scale bar: 500 µm. **c**, **d** Mortality and Smurf phenotype observed in *D. melanogaster* challenged by DSS and treated with citrus extracts for 7 d. Data are mean ± SD of *n* = 300 flies/treatment. **e**, **f** Reactive oxygen species (ROS) level and metabolic activity of *D. melanogaster* stressed with ferrous iron and treated with citrus extracts for 70 h, normalized to protein content. RFU stands for relative fluorescence units. Data are mean ± SD of *n* = 12 samples (25 flies each)/treatment. **g**, **h** Mortality and climbing performance of *D. melanogaster* stressed with ferrous iron and treated with citrus extracts for 7 d. Data are mean ± SD of *n* = 225 flies/treatment. Differences are analyzed by ordinary one-way ANOVAs with Šídák’s multiple comparison test.

Based on the in vitro experimental results, we further investigated antioxidant and intestinal barrier protective properties of AQE and its corresponding biotransformed extract FermCAE in female *D. melanogaster*. Considering the metabolic complexity of the whole organism compared to the cell culture, the effect of food matrix on bioavailability^33,34^ and the involvement of gut microbiota of *D. melanogaster* in intestinal function^35,36^, we doubled the treatment extract concentrations compared to the in vitro experiments. The dextran sulphate sodium (DSS) induced intestinal barrier challenge was performed simultaneously with AQE or FermCAE treatment (Fig. 8a, 1). DSS-induced rupture of intestinal barrier led to leakage of the blue dye through the entire fruit fly, turning them into so called Smurfs (Fig. 8b, 1). The choice of the fruit fly sex was based on the technical aspect of the experiment, such as clear visual identification of Smurfs, and was not due to the expectation of sex-specific effects. We scored and compared the mortality and visible midgut leakage (Smurf phenotype) separately, because some DSS-fed flies were not demonstrating Smurf phenotype (Fig. 8b, 2). Our findings confirmed, that DSS significantly increases mortality and induces intestinal barrier rupture compared to control fruit flies. Importantly, we observed a significant decrease of mortality in *D. melanogaster* supplemented with higher concentrations of both citrus extracts (Fig. 8c), 5% AQE (*p* = 0.0069) or 5% FermCAE (*p* < 0.0001), respectively, with FermCAE demonstrating better protective effect compared to AQE (14.43% *vs*. 8.61% of mortality reduction compared to untreated DSS-challenged group). Visible damage of intestinal barrier was reduced for fruit flies treated with both extracts in a concentration-dependent manner (Fig. 8d). Moreover, Smurf phenotype reduction was comparable in 5% AQE and 5% FermCAE treatments (both *p* < 0.0001) with 19.87% and 21.06% respectively, compared to the untreated DSS-challenged group.

Taken together, the aglycone-enriched biotransformed citrus extract demonstrated a significant protective effect in female *D. melanogaster* under induced intestinal barrier challenge and reduced mortality in stressed fruit flies.

### Biotransformed citrus extracts reduce ROS level and improve climbing performance in stressed female *D. melanogaster*

To test whether the observed antioxidant effect of biotransformed extracts in vitro could be transferred to an in vivo model, we induced oxidative stress in female *D. melanogaster* using supplementation of ferrous iron (Fe^2+^) several times above the physiological level^37,38^ (Fig. 8a, 2). ROS formation and intracellular metabolic activity were quantitated after 70 h of induced oxidative stress. In consistency with our in vitro results, citrus extracts significantly reduced ROS in fruit flies in a concentration-dependent manner (Fig. 8e). Particularly, the extract with the highest aglycone content FermCAE at a concentration of 5% showed the best antioxidant effect reducing ROS by 64.27% compared to the group with stressor only. Both concentrations of FermCAE also showed a trend towards better antioxidant activity compared to respective concentrations of AQE, with a significant difference between the 2.5% extract groups (*p* = 0.0014). No significant improvement of suppressed metabolic activity was observed in any of the stressed and extract-treated flies (Fig. 8f), compared to the untreated control group with significantly higher metabolic activity (*p* < 0.0001). All groups with ferrous iron diet demonstrated nearly equal levels of metabolic activity reduction.

In parallel to biochemical parameters, we investigated climbing activity and mortality of *D. melanogaster* due to oxidative stress. Our findings revealed that FermCAE significantly reduced mortality in flies upon oxidative stress (Fig. 8g) in a concentration-dependent manner; *p* = 0.0252 for 2.5% and *p* < 0.0001 for 5% FermCAE. Similar to in vitro experiments, a positive but much weaker effect was also observed for AQE, which was significant only for 5% AQE with *p* = 0.0002, indicating superior protective properties of biotransformed extract FermCAE. This weak positive effect of AQE completely faded when the fitness level of *D. melanogaster* was concerned. In the climbing assay, where negative geotaxis of fruit flies was tested against time, only the group on a diet with the highest concentration of FermCAE was significantly better (*p* = 0.0019) than the stressor-only group (Fig. 8h).

In conclusion, biotransformed extract with highest aglycone content showed a significant antioxidant effect upon induced oxidative stress in female *D. melanogaster*, while the protective effect of non-biotransformed extract was weak to absent.

## Discussion

In this study, we demonstrated that combination of acid hydrolysis and fermentation with the probiotic bacterium *L. plantarum* improves the cellular bioavailability and bioactivity of food grade citrus extract due to deglycosylation of its prototypic flavonoids, making this plant material well applicable in nutraceuticals, food supplements or functional food and feed.

Providing mechanistic detail and quantification, we demonstrated the superior antioxidant activity of biotransformed citrus extract in two mammalian intestinal epithelial cell lines, IPEC-J2 and Caco-2, compared to non-biotransformed citrus extracts. Moreover, we were able to show that improvement of bioactivity by biotransformation was attributed to the increase in naringenin and hesperetin levels, which were better taken up by cells than glycosylated flavonoids. Our findings are consistent with literature on flavonoid uptake in Caco-2 cells, where uptake of naringin normalized to cell protein was previously found to be low^39^. In contrast, uptake of its aglycone naringenin was comparatively higher, as confirmed in other studies^40,41^. Similarly, the transport rate of the aglycone hesperetin was also found to be superior than that of the glycoside hesperidin^42^. However, none of the aforementioned studies investigated the cellular uptake and transport of target flavonoids in a mixture within a matrix of a plant extract, in which compounds other than naringin, hesperidin and the respective aglycones were present. Noteworthy, the extracts were dissolved in HBSS for uptake and transport studies, although HBSS does not mimic the properties and composition of gastrointestinal fluids. Due to technical limitations in HPLC analysis, it was not possible to use a solvent that better simulated intestinal fluids. The necessary purification steps for removing the interfering compounds would have resulted in analyte concentrations below the detection limit.

Considering the connection between oxidative stress and inhibition of wound healing, we could also confirm enhanced cell migration and thus wound healing capacity of IPEC-J2 cells incubated with biotransformed citrus extracts. No studies were found on naringin, hesperidin and their respective aglycones in the context of our IPEC-J2 experiments.

Moreover, the superior bioactivity of fermented citrus extract over non-fermented was confirmed in *D. melanogaster*. Previous studies indicated antioxidant activities of naringin^43,44^ and hesperidin^37,45^ in fruit flies. In particular, the survival as well as climbing performance of *D. melanogaster* stressed by iron excess and treated with purified hesperidin was improved^37,45^, which is also confirmed with our results despite the slight differences in treatment duration and the way of hesperidin supplementation. The simultaneous treatment with a different oxidative stressor – trichloroethylene – and hesperidin also significantly reduced the ROS level and restored acetylcholinesterase activity in the fruit flies compared to the stressor only group^37,45^. The antioxidant effects of the glycoside naringin were as well examined with a different stressor, such as paraquat, and resulted in improved survival rate, as well as locomotor activity^43,44^. In contrast, the aglycones naringenin and hesperetin or plant extracts containing them have not been investigated in *D. melanogaster* so far. Here, we demonstrated an enhanced antioxidant capacity of biotransformed citrus extract with increased concentration of the respective aglycones fed to *D. melanogaster*. Fermented citrus extract not only reduced ROS level in chemically stressed fruit flies significantly stronger compared to non-biotransformed extract, but also led to superior reduction of stress-induced mortality.

In consensus with our in vitro cell migration experiments in intestinal epithelial cells, intestinal barrier challenge induced by DSS confirmed protective properties of biotransformed citrus extracts in vivo. DSS was proven to induce intestinal tissue damage in *D. melanogaster*^46^, but remains an underrated instrument to study the protective capacity of phytochemical compounds. It was previously shown that DSS-induced intestinal inflammation could be reduced by anthocyanins^47^. There, a reduction of intestinal barrier damage of ~20% was achieved in the simultaneous treatment of experimental flies with DSS and bilberry anthocyanins extracts compared to DSS only. Another study showed that the aglycone hesperetin ameliorated DSS-induced damage in Caco-2 cells, as well as reduces the damage in mice^48^. In the present study, we demonstrated that DSS-induced intestinal barrier damage and related mortality in *D. melanogaster* were as well reduced due to supplementation of flavonoids in form of citrus extracts (hesperetin and naringenin included) and this reduction was even greater when aglycone-enriched extracts were applied. It is important to mention that gut microbiota of living organisms can have an effect on flavonoid bioavailability. Therefore, in the conducted experiments, the intestinal bacteria of *D. melanogaster* might have influenced the intestinal deglycosylation or further metabolization of naringin and hesperidin in the fed extracts as well.

In the respective in vitro THP-1 model of inflammation, a significantly stronger reduction of pro-inflammatory cytokines could also be traced back to aglycones with naringenin expressing the maximal anti-inflammatory effect. Our findings on reduction of IL-6 and TNF-α are in line with previously investigated anti-inflammatory properties of naringenin^49^ and hesperetin^50,51^ and expands these data with the anti-inflammatory properties of the biotransformed citrus extracts that contain both aglycones. To our knowledge, we first demonstrated flavanone-related decreases of the chemokines CXCL11, CCL7, and CXCL9 in the LPS-stimulated macrophage model. In particular, we showed the superior anti-inflammatory properties of naringenin and hesperetin compared to their glycosylated forms naringin and hesperidin, respectively. Reduction of CCL7 upon naringenin treatment was once demonstrated in high-fat diet-induced obese mice^52^, while CXCL9 and CXCL11 were not investigated in context of flavanones at all.

The topic whether aglycones are more beneficial in the human body than glycosylated citrus flavonoids has been discussed extensively in literature, and to our knowledge, there is still no clear answer. For example, it has been reported that *O*-glycosylation of naringenin and hesperetin generally reduces the biological activity, such as anti-inflammatory and antioxidant properties of the compounds but can enhance other biological benefits such as anti-HIV activity, tyrosinase inhibition or anti-rotavirus activity^10^. At this point it has to be emphasized that findings from in vitro and in vivo studies are not directly applicable to humans without further investigations. Therefore, it can neither be generalized that aglycones always have a better effect on human health nor that conversion to aglycones is required to achieve a beneficial effect on health.

Given the demonstrated bioactivity of the aglycones naringenin and hesperetin, the question arises whether existing methods of flavonoid deglycosylation, such as enzymatic cleavage, acid treatment or application of genetically modified organisms, are optimal in terms of application field, sustainability and costs. It was recently demonstrated that incubation with 0.5 M hydrochloric acid in 80% ethanol effectively hydrolyzed the glycosylated flavonoid rutin to its aglycone form quercetin within 3 h^53^. However, application of hydrogen chloride is limited in food and feed technology, unlike citric acid^54^ applied in this study. Moreover, citric acid is considered more environmentally friendly than hydrogen chloride because it is metabolized to carbon dioxide and water. In our approach, a 4.5-fold (naringenin) and 3.1-fold (hesperetin) higher aglycone content was obtained by 4 h of citric acid hydrolysis and 24 h fermentation with *L. plantarum* compared to the citrus extract prepared with water. There have been previous attempts to use citric acid for deglycosylation of flavonoids^55^. The researchers increased the extraction yield of the aglycones apigenin and luteolin in celery extract by citric acid hydrolysis and treatment with the purified enzyme β-glucosidase. They could even increase the content of the aglycones luteolin and apigenin by a factor of 14.5 and 83.6, respectively. However, the biotechnological application of purified enzymes is associated with high costs, since enzyme production is energy- and labor-intensive^56^, and free enzymes cannot be conveniently reused. In this regard, immobilized enzymes offer the advantage that they can be reused for multiple runs. For example, immobilized naringinase was recently shown to retain 80% of its original activity after 10 runs^57^. While highly efficient, enzymatic cleavage, as well as application of bioengineered microorganisms also imply application limitations^58^ and heavier environmental footprint.

In our study, we presented a combination of citric acid hydrolysis with tailored LAB fermentation for conversion of glycosylated flavanones to aglycones in food grade citrus extracts. When material costs are considered, our suggested method is a cost-efficient alternative to the enzymatic deglycosylation and a sustainable technology that assists deglycosylation of flavanones. For instance, costs of citric acid, LAB strains and nutrient media for initial LAB cultivation, required for biotransformation of one unit of food grade citrus extract, are lower compared to the costs for pure naringinase, required for deglycosylation of the same weight unit, even if the enzyme will be reused multiple times. Moreover, food-grade components allow the biotransformed plant products to be used as dietary supplements, functional food and beverages, as well as additives for animal feed. Therefore, it might be of interest to use the entire fermentation product including dissolved and undissolved flavonoids as well as bacteria for large-scale applications. In this study, however, we only used the supernatant with dissolved flavonoids as final extracts for in vitro and in vivo experiments. Nevertheless, we consider our biotransformation approach a promising method to improve the bioavailability and bioactivity of plant extracts rich in flavanones.

## Methods

### Extract preparation and biotransformation of flavonoids

Citrus bioflavonoids complex 60 T0110890 (named complex 60) was purchased from Evesa Extractos Vegetales S.A (Cadiz, Spain). Complex 60, a light brown powder, consists of whole dried fruit of *Citrus aurantium* var. *amara L*., *Citrus sinensis*, and *Citrus paradisi* collected from Seville, Spain and has a total flavonoid content of > 60% with the main flavonoids identified as naringin, neohesperidin and hesperidin.

For preparation of the non-biotransformed aqueous extract (referred to as AQE), a 10% (weight per weight) solution of complex 60 in deionized water was prepared, incubated at 90 °C for 4 h and centrifuged at 4,000× *g* for 10 min. After extraction, approximately 60% of the flavonoids of complex 60 were dissolved in the supernatant. Only the supernatant was used for further experiments.

### Citric acid hydrolysis

For citric acid hydrolysis, 10 g of complex 60 were incubated with 50 ml of citric acid solution (0.25 M, 0.50 M or 1.00 M) in a water bath under constant stirring at 90 °C for 4 h. After incubation, sodium citrate solution (0.25 M, 0.50 M, 1.00 M, respectively) was added to reach a final weight of 100 g. The final citric acid hydrolyzed extract (referred to as CAE) was prepared with 0.25 M of citric acid and sodium citrate solution. Extracts were centrifuged at 4,000× *g* for 10 min to obtain the supernatants which were used for fermentation.

### Cultivation of bacteria

*L. plantarum* (DSM 20205), *L. brevis* (DSM 6235) and *L. paracasei* (DSM 20312) were obtained from the German Collection of Microorganisms and Cell Cultures GmbH (Braunschweig, Germany), while *L. rhamnosus* (NCTC 10302) was purchased from the National Collection of Type Cultures (NCTC, Salisbury, UK). All strains were cultivated microanaerobically in de Man, Rogosa and Sharpe (MRS) broth (Carl Roth GmbH, Karlsruhe, Germany) under agitation at 37 °C. Modified MRS (pH 6.5 ± 0.2) was prepared in-house by dissolving 10 g of peptone from casein, 4 g of yeast extract, 6 g dipotassium hydrogen phosphate, 5 g of diammonium hydrogen citrate and 0.04 g of manganese sulphate per liter (all Carl Roth GmbH). The medium was heat-treated by autoclaving at 121 °C for 12 min and stored at 4 °C before use.

Gram staining was performed with a Gram staining kit (Merck, Darmstadt, Germany) according to the manufacturer’s instructions. Cells were observed via light microscopy (Nikon Eclipse 80i, objective Plan Fluor 100 × 1.30; Nikon Instruments, Amsterdam, Netherlands) using differential interference contrast (DIC). Photos were taken with the Nikon DS U1 camera (5 MPx) using the NIS-Elements software (Version 5.02.01, Nikon Instruments).

### Fermentation

Fresh overnight cultures of each strain were prepared in MRS by incubating under agitation at 37 °C for 24 h. On the next day, bacteria were sedimented by centrifugation (4,000× *g*, 10 min) and resuspended in 12 ml of modified MRS with pH 6.5 to adjust the optical density at 600 nm (OD_600_) to 0.8 (corresponds approximately to 5 × 10^8^ CFU · ml^−1^). Next, 3 ml of either AQE or CAE were added. The pH value of the mixture of medium and extract was approximately 5. For non-fermented extracts, AQE and CAE were diluted in modified MRS without bacteria (abbreviations remain AQE and CAE, respectively). Culture vials were incubated under agitation at 37 °C for up to 144 h. The number of viable cells was determined using the plate spread method. Extracts were incubated at 95 °C for 10 min to inhibit bacterial enzymes. Final extracts were fermented with *L. plantarum* for 24 h, and clarified by centrifugation (4,000× *g*, 10 min) before usage as 100% stocks (referred to as FermAQE and FermCAE) for in vitro and in vivo experiments.

Changes in the flavonoid composition between start point (t = 0 h) and end of fermentation (t = 24 h) were determined by high-performance liquid chromatography with ultraviolet detection (HPLC-UV) as described in Section “Analysis of flavonoids by HPLC-UV”. For analysis, 200 µl of sample were dried at 30 °C for 2 h using a vacuum concentrator (Labconco™ CentriVap™; Fisher Scientific GmbH, Schwerte, Germany) and redissolved with 200 µl of DMSO and 1800 µl of HPLC solvent (50% of acetonitrile in deionized water). Redissolved samples were centrifuged at 17,000× *g* for 10 min and diluted 1:10 in deionized water.

To determine precipitated flavonoids after centrifugation, the pellet (from 250 µl sample) was redissolved in 65 µl of DMSO and 185 µl of 0.1% formic acid in methanol/50% acetone, centrifuged at 17,000× *g* at 4 °C for 10 min and analyzed by HPLC.

### Enzymatic hydrolysis by α-L-rhamnosidase and β-D-glucosidase

For enzymatic hydrolysis of flavonoids, AQE was diluted 200-fold in 100 mM sodium phosphate buffer with pH 6.5 and incubated with either a recombinant α-L-rhamnosidase from prokaryotic source (1 U · ml^−1^; Megazyme, Wicklow, Ireland) or β-D-glucosidase from almonds (1 U · ml^−1^; Merck) at 37 °C for up to 120 min. Negative controls were prepared without enzymes. Samples were taken every 30 min, immediately incubated at 95 °C for 5 min to inhibit enzymatic activity and clarified using centrifugation at 17,000× *g* and 4 °C for 15 min. Supernatants were analyzed using HPLC-UV as described in Section “Analysis of flavonoids by HPLC-UV”.

### Analysis of flavonoids by HPLC-UV

Reversed phase chromatography was conducted using a Vanquish™ Core HPLC System comprised of a quaternary pump, a split sampler, a diode array detector, and a temperature-controlled column compartment (all Vanquish™) equipped with the Chromeleon™ software (Version 7.3.1, Thermo Scientific™ Dionex™, Waltham, MA, USA). Analyte separation was performed on an Accucore™ C18 column (150 mm × 3.0 mm inner diameter, 2.6 µm particle size; Thermo Scientific). The column temperature was set to 40 °C, and the injection volume was 10 µl. UV-detection was performed at wavelength 260 nm. Analytes were separated by gradient elution, with mobile phase A containing 0.1% formic acid (FA) in water, and mobile phase B containing 0.1% FA in acetonitrile, at a rate of 0.5 ml · min^−1^ (FA and acetonitrile of analytical grade). The elution gradient starting conditions were 95% A and 5% B. After 5 min of equilibration time, the starting conditions were maintained for 2 min before the proportion of B was increased to 20% at 5 min, to 30% at 15 min, to 40% at 20 min, to 60% at 23 min, and to 80% at 25 min. The last condition was held for 3 min, followed by reducing the proportion of B to 5% within 2 min. Finally, the condition with 95% A and 5% B was maintained for 10 min. The internal standards narirutin, naringin, naringenin-7-*O*-glucoside, hesperidin, neohesperidin, hesperetin-7-*O*-glucoside, naringenin (all Extrasynthese, Genay, France) and hesperetin (Sigma-Aldrich, Schnelldorf, Germany) were used for calibration in a linear range. Detailed information on calibration curves, linearity, limit of detection (LOD) and limit of quantification (LOQ) are provided in Supplementary Table 2 and Supplementary Figure 9.

### Cell culture maintenance

All chemicals and reagents were obtained from Sigma-Aldrich, if not stated otherwise. Porcine IPEC-J2 (ACC 701) and human Caco-2 (ACC 169) cells were purchased from German Collection of Microorganisms and Cell Cultures GmbH, while human THP-1 (TIB-202™) monocytes were obtained from American Type Culture Collection (Manassas, VA, USA). IPEC-J2 cells were maintained in Dulbecco’s Modified Eagle’s Medium (DMEM), Caco-2 cells in MEM Eagle with non-essential amino acids and THP-1 cells in RPMI-1640 medium, all supplemented with 10% fetal bovine serum (FBS), 100 µg · ml^−1^ penicillin and 100 µg · ml^−1^ streptomycin (all PAN-Biotech, Aidenbach, Germany). Growth medium for THP-1 cells was additionally enriched with 0.05 mM of 2‐mercaptoethanol (PAN-Biotech), as recommended by ATCC. Cells were grown at 37 °C in a humidified atmosphere (≥95%) with 5% CO_2_.

### Cell viability assay

Toxic effects of test extracts and flavanones were evaluated using a resazurin-based in vitro toxicology assay kit according to the manufacturer’s instructions. Briefly, cells were seeded into black 96‐well plates at a density of 2 × 10^4^ (IPEC-J2), 1.5 × 10^5^ (Caco-2) or 10^5^ (THP-1) cells per well, grown to 90% confluency, and incubated with test substances in growth medium for 20 min (IPEC-J2 and Caco-2) or 24 h (THP-1) at 37 °C. Then, medium was aspirated, and cells were incubated with 10% resazurin in growth medium for 1.5 h. The amount of resorufin was measured using a microplate reader in fluorescence mode (544 nm excitation, 590 nm emission; POLARstar Omega, BMG LABTECH, Ortenberg, Germany). Data were analyzed using the Omega MARS Data analysis software package (BMG LABTECH). Cell viability was normalized to untreated cells (control) grown under the same conditions.

### Transport and uptake tests in vitro

For uptake studies, Caco-2 cells were seeded in 6 cm dishes at 4.4 × 10^6^ cells per dish and grown overnight. Cells were differentiated on day 2 using DMEM supplemented with 100 µg · ml^−1^ penicillin and 100 µg · ml^−1^ streptomycin, 0.1% MITO+ Serum Extender (Corning, FAL-355006) and 5 mM butyric acid, which was refreshed on the third day. Cell layers acquired a differentiated phenotype, which was observed as the formation of domes under the microscope^59^. Differentiated Caco-2 cells were washed with HBSS and incubated with 5 ml of AQE or FermCAE (2.5% in HBSS) for 4 h at 37 °C. Then, the supernatant was removed, cell layers were washed twice with Dulbecco’s phosphate buffered saline (DPBS; PAN-Biotech) and detached using a cell scraper. After centrifugation and removal of DPBS, cell pellets were resuspended in 50% acetonitrile and placed into the ultrasonic bath for 15 min to break up the cells. For precipitation of cellular proteins, the suspensions were frozen at −20 °C for 30 min. Samples were centrifuged and the obtained supernatants were analyzed by HPLC to quantitate flavonoids taken up by the cells. The remaining pellets were dissolved in 1 M NaOH, and the protein content was determined by Quick Start™ Bradford 1× Dye Reagent (Bio-Rad Laboratories, CA, USA) according to the manufacturer’s protocol.

For transport studies Caco-2 cells were seeded at 1.65 × 10^5^ cells per trans-well insert (Thin-Cert, 0.336 cm^2^, 0.4 µm pore size; Greiner-Bio One, GRE-662640) and grown overnight. Cell layers were differentiated as described before and cell layer integrity was assessed by TEER measurement with a Millicell-ERS-2 Voltohmmeter (Merck, Darmstadt, Germany). For the experiment, differentiated cells were washed twice with HBSS and the inserts were placed in a fresh microtiter plate prefilled with 1040 µl HBSS per well. Cell layers were provided with 300 µl of AQE or FermCAE (2.5% in HBSS) in the apical compartment and incubated for 4 h at 37 °C. To ensure cell layer integrity is maintained throughout the experiment, TEER measurements were performed directly after the treatment (t = 0 h) and at the end of the transport test (t = 4 h). Flavonoid concentrations were determined by HPLC analysis as described in Section “Analysis of flavonoids by HPLC-UV”.

### ROS quantification assay in vitro

Cells were seeded into black 96‐well plates at a density of 2 × 10^4^ (IPEC-J2) or 1.5 × 10^5^ (Caco-2) cells per well and grown overnight. Next, cells were washed with DPBS and incubated simultaneously with 50 µM of the ROS probe 2’,7’-dichlorodihydrofluorescein diacetate (H_2_DCFDA)^60^ and extracts (1.25% and 2.5% in HBSS) or flavonoids (150 µM of naringin, naringenin, hesperidin or hesperetin in HBSS). Quercetin (20 µM) was used as an antioxidant positive control. Flavonoid stocks were prepared in DMSO, but concentrations applied to cells did not exceed 0.1% of DMSO. After incubation for 20 min, cells were washed twice with DPBS and stressed with 100 µM of tert-butyl hydroperoxide (tBHP; for IPEC-J2) or 500 µM of 2,2’-azobis(2-methylpropionamidine) dihydrochloride (AAPH; for Caco-2). Two different stressors were used because not every cell line responds to each stressor. Cells treated with HBSS were used as control. The amount of produced dichlorodihydrofluorescein (DCF) was determined with a microplate reader in fluorescence mode (485 nm excitation, 520 nm emission; POLARstar Omega, BMG LABTECH). Data were analyzed using the Omega MARS Data analysis software package (BMG LABTECH). Measurements started immediately after stressor addition at 0, 15, 30, 60 and 90 min. This oxidative status over time was summarized by calculation of the area under the curve normalized to the stressor treatment.

### Cell migration assay in IPEC-J2 cells

The effect of biotransformed citrus extracts on migration of stressed IPEC-J2 cells was assessed using a scratch assay^20^. IPEC-J2 cells were seeded in 24-well imaging plates with cover glass (MoBiTec, Goettingen, Germany) at a density of 2.5 × 10^5^ cells per well and grown overnight. When confluency was achieved, cells were pretreated with the extracts at a concentration of 1.25% for 6 h. Then, a straight scratch was generated with constant pressure, speed and angle using a 100 μl plastic pipette tip. Excess debris was removed by washing with DPBS. Cells were stressed with 100 µM of tBHP while incubated with the extracts. Extract treatments were compared to cells stressed with tBHP only. Untreated cells were used as control. For scanning of the scratched area, automated brightfield imaging was performed every 15 min for 420 min on an epi-fluorescent microscope (Nikon Eclipse Ti2, Tokyo, Japan) using a 2× CFI Plan Apochromat objective (NA = 0.1) with additional 1.5× magnification. For temperature and CO_2_ control, the microscope was equipped with a cage incubator (Okolab, Shanghai, China) allowing imaging of cell culture at 37 °C and 5% CO_2_. NIS-Elements software package (Version 5.02.01, Nikon, Tokyo, Japan) enabled definition of acquisition positions with respect to the scratch in each well. For scanning of multiple stage positions in one experiment, the microscope was further equipped with a x-y-stage (CMR-STG-MHIX2-motorized table, Märzhäuser, Germany) and a sCMOS camera (Zyla 4.2, Andor, Northern Ireland). Image analysis was carried out using ImageJ and the *Wound_healing_size_tool.ijm* plugin^61^. Cell front velocity (μm · h^−1^) was calculated as indicated in Eq. 1:

1 $${cell\; front\; velocity}=\frac{{wound\; closure\; speed}}{{length\; of\; cell\; front}\times 2}$$

with *wound closure speed* calculated from the slope of the linear trend line (area *vs*. time; standardized to 15−420 min) and the *length of cell front × 2* corresponded to the length of the gap area considering two cell fronts that migrate towards each other (see also *ibidi-cells in focus*^62^).

### LPS challenge assay in THP-1 cells

THP-1 cells were seeded in 6-well plates at a density of 2.75 × 10^6^ cells per well and stimulated with 50 ng · ml^−1^ of phorbol-12-myristate-13-acetate (PMA) for differentiation into macrophages. After incubation for 48 h, depleted medium was aspirated, and adherent macrophages were incubated simultaneously with 250 ng · ml^−1^ of LPS and extracts (1.25% or 2.5%) in growth medium with 1% of FBS (in total 2.5 ml per well). Cell culture supernatants containing secreted cytokines were harvested after 24 h and stored at −80 °C prior to analysis. Supernatants of extract treated cells were compared to the ones of cells challenged with LPS only. Untreated cells were used as control.

### Cytokine array analysis

The expression profile of cytokines, including chemokines, and growth factors was assessed using Human XL Cytokine Array Kit (Proteome Profiler™ Array, Bio-Techne Ltd., Abingdon, UK) following the manufacturer’s instructions. Briefly, the membranes were blocked at room temperature (RT) for 60 min and incubated with samples at 4 °C overnight. After washing, the membranes were treated with biotinylated detection antibodies at RT for 90 min. Finally, they were washed and incubated with streptavidin-horseradish peroxide at RT for 30 min. Captured proteins were visualized by incubation with chemiluminescent detection reagents for 1 min, followed by measuring the luminescence using a ChemiDoc MP Imaging System (Bio-Rad Laboratories). Semi-quantitative comparison was performed using the Image Lab Software (Bio-Rad Laboratories). The pixel intensity in each spot of the array was analyzed and the background signal was subtracted from each spot.

### Cytokine multiplex immunoassay

Based on cytokine membrane array results, the selected cytokines CCL7, CXCL11, CXCL9, IL-6, and TNF-α were quantified with Luminex xMAP® custom multiplex assay (Bio-Techne Ltd.). For this purpose, cell culture supernatants of LPS-stimulated THP-1 cells were applied undiluted for CCL7, CXCL11, CXCL9 and diluted 1:100 for IL-6 and TNF-α quantitation, respectively. Samples or standards were incubated for 2 h in presence of antibody-coated magnetic microbeads, followed by washing step (Fig. 7a). This and all the next incubation periods were performed in a sealed, light-protected black well plate agitated with thermo-shaker at RT, 800 rpm. For the next 1 h of incubation, biotinylated detection antibodies were added. Microbeads with analyte sandwiches were then washed and incubated with streptavidin-phycoerythrin solution for 30 min. Afterwards, unbound reagent was removed. Prepared samples were resuspended with washing buffer, acquired by Luminex® 200™ and analyzed with xPonent® acquisition software, Version 4.3 (both Luminex Corp., Austin, TX, USA) in technical duplicates. Calibration and interpolation of unknown concentrations was performed with weighted logistic 5P curve with intra-assay goodness-of-fit R^2^ = 0.995. Intra-assay coefficient of variation equaled 5.26%, intra-assay recovery of standards equaled 100.53%. Detailed inter-assay characteristics per analyte are listed in Supplementary Table 3.

### Husbandry of *D. melanogaster* and preparation of experimental fruit flies

Parental fruit flies *D. melanogaster*, strain *w*^*1118*^ (University of Kiel, Kiel, Germany; strain no. 5905 Bloomington *Drosophila* Stock Center, Bloomington, IN, USA), were reared in our laboratory for over 60 generations in standardized conditions at 25 °C, 60% relative humidity (RH), 12:12 h light-dark cycle. Controlled climate conditions were insured by climate chamber HPP750 eco (Memmert GmbH + Co. KG, Germany). If not stated otherwise, all reagents in the *D. melanogaster* experiments were purchased from Carl Roth GmbH. To prepare aged-synchronized fruit flies, parental flies were transferred to the embryo collection cages with Nutri-Fly® grape agar petri dishes (both Genesee Scientific, San Diego, CA, USA) prepared according to the manufacturer’s instructions. To induce egg laying behavior, parental flies were provided with fresh yeast paste consisting of active dry yeast “Red star” (Genesee Scientific) and deionized water. After 24 h, the petri dish was replaced with a fresh one. In ca. 20 h, eggs were collected from grape agar, washed with sterile DPBS 3 times, and dispensed on larval growth medium (35 µl per stock bottle). In 9 d freshly eclosed synchronized adults were transferred to the stock bottles with sugar-yeast medium and allowed to mate for 2 d before the sorting to sexes. The medium for larval growth was prepared as described^22,63^ from 1% agar, 5.5% glucose, 3% sucrose, 2.5% inactive dry yeast, 6% yellow cornmeal (both Genesee Scientific), all percentage is weight per volume. Sugar-yeast medium consisted of 1.5% agar, 5% sucrose, 10% inactive dry yeast (weight per volume). Both media were preserved with 1% of methyl 4-hydroxybenzoate solution (10% solution in absolute ethanol, weight per volume), and 0.48% propionic acid (volume per volume).

### Intestinal barrier challenge in *D. melanogaster*

To investigate the protective effect of biotransformed citrus extracts in vivo, a series of three experiments with DSS challenge were conducted. The experiments were based on the method of Amcheslavsky et al.^46^ with our modifications^22^ (Fig. 8a, 1). 5 days old (d. o.) *w*^*1118*^ female *D. melanogaster* were sorted to groups of 25 ± 2 adult flies per vial and transferred to non-nutritional medium (1.5% agar in water) in order to prevent drying out of the experimental media in the empty vials. Experimental liquid media consisted of aqueous solution of 5% sucrose, 1% Brilliant Blue FCF (C.I. 42090), 5% DSS with average MW 40,000 g · mol^−1^, and either 2.5% or 5% of AQE or FermCAE, and were introduced on a piece of 1.5 × 3 cm, 1.5 mm thick gel blotting paper (Whatman, Cytiva, UK). The control group was fed with aqueous solution of 5% sucrose and 1% Brilliant Blue FCF only. Each experimental condition was represented by 4 vials (≈100 fruit flies) per experiment. These experimental flies were maintained in standard climate conditions at 25 °C and 60% RH for 7 d and were transferred to a fresh agar vial with a soaked filter paper 4 times per experiment. The number of dead flies as well as of flies with Smurf phenotype was scored daily. Female *D. melanogaster* were scored as Smurfs as described by Rera et al.^64^ when they demonstrated blue coloration observed outside intestinal area, e.g. in thorax and limbs (Fig. 8b, 1).

### Induction of oxidative stress in *D. melanogaster*

To evaluate possible antioxidant effects of the biotransformed extract FermCAE in vivo, series of twelve experiments were conducted using ferrous iron as oxidative stressor. For that 5 d. o. *w*^*1118*^ female *D. melanogaster* were sorted to groups of 25 ± 2 adult flies per vial and placed on solid experimental media that was exchanged every 2−3 d. Experimental media were based on sugar-yeast medium described above (also applied as control group) with addition of 30 mM iron (II) sulphate heptahydrate as oxidation-inducing stressor^37,65^ and 2.5% or 5% of AQE or FermCAE in one of the 4 treatment groups, respectively (Fig. 8a, 2). Each experimental group was represented with ≈ 100 flies (4 vials) per experiment. With respect to physiological changes related to circadian rhythm^66–68^ fruit flies were placed on experimental media at the exact same day time (11:30 am) in each of 3 experiments for biochemical assays or for survivorship and climbing performance.

### ROS quantification and metabolic activity assays in *D. melanogaster*

For both biochemical assays, female *D. melanogaster* were sacrificed exactly after 70 h. Homogenizer tubes with stainless steel beads (Precellys Lysing Kit for Hard Tissue Grinding, Bertin Technologies, Main, Germany) were filled with 300 µl of ice-cold DPBS containing 1 µl · ml^−1^ protease inhibitor cocktail (PIC; Sigma-Aldrich). Flies were anesthetized with CO_2_ for 10 s, transferred with a funnel to the homogenizer tubes and homogenized in Precellys Evolution tissue homogenizer (Bertin Technologies) at 6000 rpm for 40 s. Afterwards, 700 µl of DPBS-PIC mix were added to homogenate on ice. 800 µl of sample were transferred to the fresh tubes without steel beads and centrifuged for 10 min at 4 °C and 14,000× *g*. Clear supernatant was kept on ice and assayed immediately in ROS quantification and metabolic activity assays, as well as in Bradford assay for protein normalization. For ROS quantification, 90 µl of DPBS, 100 µl of samples and 10 µl of 100 µM H_2_DCFDA were incubated in a black 96‐well plate light-protected for 2 h. Additionally blanks of homogenization buffer with H_2_DCFDA and sample supernatants without H_2_DCFDA were tested to exclude autofluorescence interference. End point measurement of fluorescent DCF product was performed as described in Section “ROS quantification assay in vitro”. The metabolic activity assay was based on reduction of resazurin to fluorescent resorufin by NADH, NADPH, or FADH. In brief, 50 µl of sample and 50 µl of 25 µM resazurin solution (Thermo Fisher Scientific) were incubated light-protected for 2 h. Analogue to ROS assay, blanks were tested to exclude variating autofluorescence of samples or homogenization buffer. End point measurement of fluorescent resorufin was performed as described in Section “Cell viability assay”. To normalize obtained values of biochemical assays, proteins in sample supernatants were quantified with Bradford assay^69^ with modifications. Obtained supernatants were diluted 1:20 in 20 mM sodium chloride solution and assayed with Quick Start™ Bradford 1× Dye Reagent (Bio-Rad Laboratories) in 1:5 proportion. Calibration was performed with 2-fold dilution of BSA solutions (Sigma-Aldrich) in concentrations from 0 to 100 µg · ml^−1^. After 1 h incubation, absorbance was measured with POLARstar® Omega Microplate Reader at 590 nm and normalized at 450 nm. The second wavelength was used for linearization of calibration graph as suggested by Ernst&Zor^70^. All fluorescence and absorbance measurements were processed with Omega Software, Version 2.10 and Mars Data Analysis Software, Version 2.30 (both BMG LABTECH GmbH). All measurements were performed in technical duplicates except Bradford assays, which were run in technical triplicates.

### Mortality and climbing performance experiments in *D. melanogaster*

To further investigate physiological effects of citrus bioflavonoids on fruit flies under the oxidative challenge, 5 d. o. *w*^*1118*^ female *D. melanogaster* flies were reared on experimental media with ferrous iron for 7 d. During that period *D. melanogaster* were transferred to fresh media 4 times, dead flies were scored during every transfer. Results were expressed as percent of dead insects from all fruit flies in each vial. Climbing activity of *D. melanogaster* was assessed as previously described^71,72^ with minor modifications: on day 7 percent of subjects, climbed to the top of the vial (5 cm distance) in 20 s after being tapped to the bottom, was scored. For each vial, the measurement was performed twice, and the mean value was obtained.

### Statistics, reproducibility and visual content

All statistical tests were performed in GraphPad Prism, Version 9.4.1 (GraphPad Software, San Diego, CA, USA) as recommended in GraphPad Statistics Guide. Prior to significance tests each data set was tested to normality of data distribution with Shapiro-Wilk test. Normally distributed data were analyzed with two-tailed t-test (for 2 data sets) or ordinary one-way ANOVA (for > 2 data sets). Dependent on the research question, ANOVA was extended with one of the following multiple comparison tests: 1) comparison of all data sets with each other—Tukey’s test, 2) comparison of treatment group vs. control or stressor group—Dunnett’s test, 3) comparison of selected groups with control, stressor, or each other — Šídák’s test. Data that did not pass normality distribution test were analyzed with Kruskal-Wallis’s test with Dunn’s multiple comparisons test (for > 2 data sets). All tests were conducted with 95% confidence interval (CI). Detailed summaries of statistical tests including number of subjects in each group *n*, F-, H- or t-values, as well as degree of freedom are provided in Supplementary Tables 4−6. *p* values ≤ 0.05 were considered significant and indicated as * (*p* ≤ 0.05), ** (*p* ≤ 0.01), *** (*p* ≤ 0.001) or **** (*p* ≤ 0.0001). The uncertainty of the visualized data was expressed via standard deviations (SD) shown as error bars. All graphs were constructed in GraphPad Prism, Version 9.4.1. Schematic overviews of respective methods were created by authors in BioRender application (Science Suite Inc., Toronto, ON, Canada). Images and photos were processed in CorelDRAW® Graphics Suite, Version 21.0.0.593 (Corel Corporation, Ottawa, ON, Canada).

### Reporting summary

Further information on research design is available in the Nature Portfolio Reporting Summary linked to this article.

### Supplementary information

Supplementary information

Description of Supplementary Materials

Supplementary Data

Supplementary Movie

Reporting summary

## Data Availability

Numerical source data for all charts and graphs can be found in Supplementary Data. Further data that support the findings of this study are not openly available due to reasons of sensitivity and are available from the corresponding author upon reasonable request.

## References

[1] Guan R (2021). A review of dietary phytochemicals and their relation to oxidative stress and human diseases. Chemosphere.

[2] Liu S (2022). Review of phytochemical and nutritional characteristics and food applications of Citrus L. fruits. Front. Nutr..

[3] Zhang M, Zhu S, Yang W, Huang Q, Ho C-T (2021). The biological fate and bioefficacy of citrus flavonoids: bioavailability, biotransformation, and delivery systems. Food Funct..

[4] Wang Y (2021). Citrus flavonoids and their antioxidant evaluation. Crit. Rev. Food Sci. Nutr..

[5] Barreca D (2017). Flavanones: citrus phytochemical with health-promoting properties. BioFactors.

[6] Ren H (2016). Hesperetin suppresses inflammatory responses in lipopolysaccharide-induced RAW 264.7 cells via the Inhibition of NF-κB and activation of Nrf2/HO-1 pathways. Inflammation.

[7] Pinho-Ribeiro FA (2016). The citrus flavonone naringenin reduces lipopolysaccharide-induced inflammatory pain and leukocyte recruitment by inhibiting NF-κB activation. J. Nutr. Biochem..

[8] Stevens Y (2019). The intestinal fate of citrus flavanones and their effects on gastrointestinal health. Nutrients.

[9] Park C-M, Kim G-M, Cha G-S (2021). Biotransformation of flavonoids by newly isolated and characterized *Lactobacillus pentosus* NGI01 strain from kimchi. Microorganisms.

[10] Xiao J (2017). Dietary flavonoid aglycones and their glycosides: Which show better biological significance?. Crit. Rev. Food Sci. Nutr..

[11] Di Majo D (2005). Flavanones in citrus fruit: structure–antioxidant activity relationships. Food Res. Int..

[12] Nielsen ILF (2006). Bioavailability is improved by enzymatic modification of the citrus flavonoid hesperidin in humans. A randomized, double-blind, crossover trial. J. Nutr..

[13] Da Silva CMG (2013). Enhancement of the antioxidant activity of orange and lime juices by flavonoid enzymatic de-glycosylation. Food Res. Int..

[14] FDA. GRAS Notices. Available at https://www.cfsanappsexternal.fda.gov/scripts/fdcc/index.cfm?set=GRASNotices&sort=GRN_No&order=DESC&startrow=1&type=basic&search=plantarum (2022).

[15] Koutsoumanis K (2021). Update of the list of QPS-recommended biological agents intentionally added to food or feed as notified to EFSA 14: suitability of taxonomic units notified to EFSA until March 2021. EFSA J..

[16] Mueller M (2018). Rhamnosidase activity of selected probiotics and their ability to hydrolyse flavonoid rhamnoglucosides. Bioprocess Biosyst. Eng..

[17] Pereira-Caro G (2018). Catabolism of citrus flavanones by the probiotics *Bifidobacterium longum* and *Lactobacillus rhamnosus*. Eur. J. Nutr..

[18] Müller U (2018). In vitro and in vivo inhibition of intestinal glucose transport by guava (*Psidium guajava*) extracts. Mol. Nutr. Food Res..

[19] Blank-Landeshammer B (2022). Improved bioavailability and bioaccessibility of lutein and isoflavones in cultured cells in vitro through interaction with ginger, curcuma and black pepper extracts. Antioxidants.

[20] Vergauwen H (2015). Trolox and ascorbic acid reduce direct and indirect oxidative stress in the IPEC-J2 cells, an in vitro model for the porcine gastrointestinal tract. PLoS one.

[21] Cao S (2021). AMPK-PINK1/Parkin mediated mitophagy is necessary for alleviating oxidative stress-induced intestinal epithelial barrier damage and mitochondrial energy metabolism dysfunction in IPEC-J2. Antioxidants.

[22] Heckmann M (2022). Extracts prepared from feed supplements containing wood lignans improve intestinal health by strengthening barrier integrity and reducing inflammation. Molecules.

[23] Chanput W, Mes JJ, Wichers HJ (2014). THP-1 cell line: an in vitro cell model for immune modulation approach. Int. Immunopharmacol..

[24] Staats S, Lüersen K, Wagner AE, Rimbach G (2018). *Drosophila melanogaster* as a versatile model organism in food and nutrition research. J. Agric. Food Chem..

[25] Wong ACN, Vanhove AS, Watnick PI (2016). The interplay between intestinal bacteria and host metabolism in health and disease: lessons from *Drosophila melanogaster*. Dis. Model. Mech..

[26] Capo F, Wilson A, Di Cara F (2019). The intestine of *Drosophila melanogaster*: an emerging versatile model system to study intestinal epithelial homeostasis and host-microbial interactions in humans. Microorganisms.

[27] Sandner G, König A, Wallner M, Weghuber J (2022). Alternative model organisms for toxicological fingerprinting of relevant parameters in food and nutrition. Crit. Rev. Food Sci. Nutr..

[28] Le Bourg E (2001). Oxidative stress, aging and longevity in *Drosophila melanogaster*. FEBS Lett..

[29] Seto NO, Hayashi S, Tener GM (1990). Overexpression of Cu-Zn superoxide dismutase in *Drosophila* does not affect life-span. Proc. Natl Acad. Sci. USA.

[30] Orr WC, Arnold LA, Sohal RS (1992). Relationship between catalase activity, life span and some parameters associated with antioxidant defenses in *Drosophila melanogaster*. Mech. Ageing Dev..

[31] Beekwilder J (2009). Characterization of rhamnosidases from *Lactobacillus plantarum* and *Lactobacillus acidophilus*. Appl. Environ. Microbiol..

[32] Kurahashi T, Fujii J (2015). Roles of antioxidative enzymes in wound healing. J. Dev. Biol..

[33] Kamiloglu S, Tomas M, Ozdal T, Capanoglu E (2021). Effect of food matrix on the content and bioavailability of flavonoids. Trends Food Sci. Technol..

[34] Dima C, Assadpour E, Dima S, Jafari SM (2020). Bioavailability of nutraceuticals: role of the food matrix, processing conditions, the gastrointestinal tract, and nanodelivery systems. Compr. Rev. Food Sci. Food Saf..

[35] Miguel-Aliaga I, Jasper H, Lemaitre B (2018). Anatomy and physiology of the digestive tract of *Drosophila melanogaster*. Genetics.

[36] Lesperance DNA, Broderick NA (2020). Gut bacteria mediate nutrient availability in *Drosophila* diets. Appl. Environ. Microbiol..

[37] Poetini MR (2018). Hesperidin attenuates iron-induced oxidative damage and dopamine depletion in *Drosophila melanogaster* model of Parkinson’s disease. Chem. Biol. Interact..

[38] Jimenez-Del-Rio M, Guzman-Martinez C, Velez-Pardo C (2010). The effects of polyphenols on survival and locomotor activity in *Drosophila melanogaster* exposed to iron and paraquat. Neurochem. Res..

[39] Turner MD, Nedjai B, Hurst T, Pennington DJ (2014). Cytokines and chemokines: At the crossroads of cell signalling and inflammatory disease. Biochim. Biophys. Acta.

[40] Zhang Z-D (2022). Uptake and transport of naringenin and its antioxidant effects in human intestinal epithelial Caco-2 cells. Front. Nutr..

[41] Nait Chabane M, Al Ahmad A, Peluso J, Muller CD, Ubeaud G (2009). Quercetin and naringenin transport across human intestinal Caco-2 cells. J. Pharm. Pharmacol..

[42] Kobayashi S, Tanabe S, Sugiyama M, Konishi Y (2008). Transepithelial transport of hesperetin and hesperidin in intestinal Caco-2 cell monolayers. Biochim. Biophys. Acta.

[43] Lashmanova E (2017). The Evaluation of geroprotective effects of selected flavonoids in *Drosophila melanogaster* and *Caenorhabditis elegans*. Front. Pharmacol..

[44] Anh NTT, Nishitani M, Harada S, Yamaguchi M, Kamei K (2011). A *Drosophila* model for the screening of bioavailable NADPH oxidase inhibitors and antioxidants. Mol. Cell. Biochem..

[45] Abolaji AO, Babalola OV, Adegoke AK, Farombi EO (2017). Hesperidin, a citrus bioflavonoid, alleviates trichloroethylene-induced oxidative stress in *Drosophila melanogaster*. Environ. Toxicol. Pharmacol..

[46] Amcheslavsky A, Jiang J, Ip YT (2009). Tissue damage-induced intestinal stem cell division in *Drosophila*. Cell Stem Cell.

[47] Zhang G, Gu Y, Dai X (2022). Protective effect of bilberry anthocyanin extracts on dextran sulfate sodium-induced intestinal damage in *Drosophila melanogaster*. Nutrients.

[48] Zhang J, Lei H, Hu X, Dong W (2020). Hesperetin ameliorates DSS-induced colitis by maintaining the epithelial barrier via blocking RIPK3/MLKL necroptosis signaling. Eur. J. Pharmacol..

[49] Bodet C, La VD, Epifano F, Grenier D (2008). Naringenin has anti-inflammatory properties in macrophage and ex vivo human whole-blood models. J. Periodontal Res..

[50] Xagorari A (2001). Luteolin inhibits an endotoxin-stimulated phosphorylation cascade and proinflammatory cytokine production in macrophages. J. Pharmacol. Exp. Ther..

[51] Comalada M (2006). Inhibition of pro-inflammatory markers in primary bone marrow-derived mouse macrophages by naturally occurring flavonoids: analysis of the structure-activity relationship. Biochem. Pharmacol..

[52] Tsuhako R, Yoshida H, Sugita C, Kurokawa M (2020). Naringenin suppresses neutrophil infiltration into adipose tissue in high-fat diet-induced obese mice. J. Nat. Med..

[53] Yang J (2019). Conversion of rutin to quercetin by acid treatment in relation to biological activities. Prev. Nutr. Food Sci..

[54] Scientific opinion on the safety and efficacy of citric acid when used as a technological additive (preservative) for all animal species. *EFSA J*. **13**, (2015). 10.2903/j.efsa.2014.3827 PMC13136929 42087943

[55] Che DN (2021). Citric acid and enzyme-assisted modification of flavonoids from celery (*Apium graveolens*) extract and their anti-inflammatory activity in HMC-1.2 cells. J. Food Biochem..

[56] Puri M, Banerjee UC (2000). Production, purification, and characterization of the debittering enzyme naringinase. Biotechnol. Adv..

[57] Bodakowska-Boczniewicz J, Garncarek Z (2020). Immobilization of naringinase from *Aspergillus niger* on a magnetic polysaccharide carrier. Molecules.

[58] Leonard W, Zhang P, Ying D, Adhikari B, Fang Z (2021). Fermentation transforms the phenolic profiles and bioactivities of plant-based foods. Biotechnol. Adv..

[59] Marziano M (2019). Monitoring Caco-2 to enterocyte-like cells differentiation by means of electric impedance analysis on printed sensors. Biochim. Biophys. Acta Gen. Subj..

[60] Wang H, Joseph JA (1999). Quantifying cellular oxidative stress by dichlorofluorescein assay using microplate reader. Free Radic. Biol. Med..

[61] Suarez-Arnedo A (2020). An Image J plugin for the high throughput image analysis of in vitro scratch wound healing assays. PloS one.

[62] ibidi cells in focus. Experimental Setup Optimization and Data Analysis of Wound Healing Assays. Application Note 30, Version 1.3. Available at https://ibidi.com/img/cms/support/AN/AN30_Wound_Healing_Data_Analysis.pdf (2019).

[63] Piegholdt S, Rimbach G, Wagner AE (2016). Effects of the isoflavone prunetin on gut health and stress response in male *Drosophila melanogaster*. Redox Biol..

[64] Rera M, Clark RI, Walker DW (2012). Intestinal barrier dysfunction links metabolic and inflammatory markers of aging to death in *Drosophila*. Proc. Natl Acad. Sci. USA.

[65] Jomova K, Valko M (2011). Advances in metal-induced oxidative stress and human disease. Toxicology.

[66] Xu K, Zheng X, Sehgal A (2008). Regulation of feeding and metabolism by neuronal and peripheral clocks in *Drosophila*. Cell Metab..

[67] Dubowy C, Sehgal A (2017). Circadian rhythms and sleep in *Drosophila melanogaster*. Genetics.

[68] Zhang, Y. & Emery, P. Molecular and neural control of insect circadian rhythms. *Insect Biochem. Mol. Biol*. (Elsevier), 513–551 (2012).

[69] Bradford MM (1976). A rapid and sensitive method for the quantitation of microgram quantities of protein utilizing the principle of protein-dye binding. Anal. Biochem..

[70] Ernst O, Zor T (2010). Linearization of the Bradford protein assay. J. Vis. Exp..

[71] Chambers RP (2013). Nicotine increases lifespan and rescues olfactory and motor deficits in a *Drosophila* model of Parkinson’s disease. Behav. Brain Res..

[72] Bartholomew NR, Burdett JM, VandenBrooks JM, Quinlan MC, Call GB (2015). Impaired climbing and flight behaviour in *Drosophila melanogaster* following carbon dioxide anaesthesia. Sci. Rep..

