# Supplementary material for: Combined acid hydrolysis and fermentation improves bioactivity of citrus flavonoids in vitro and in vivo
Source: Commun Biol. 2023 Oct 25;6:1083. doi: 10.1038/s42003-023-05424-7 (PMC10600125; doi:10.1038/s42003-023-05424-7)
Supplement: Supplementary file 1 — Supplementary information [file 42003_2023_5424_MOESM1_ESM.pdf]

Supplementary Information for

Combined acid hydrolysis and fermentation improves bioactivity of citrus flavonoids *in vitro* and *in vivo*

Alice König[1,2*], Nadiia Sadova[1*], Marion Dornmayr[1,2], Bettina Schwarzinger[1,2], Cathrina Neuhauser[1], Verena Stadlbauer[1,2], Melanie Wallner[1,2], Jakob Woischitzschläger[1], Andreas Müller[3], Rolf Tona[3], Daniel Kofel[3] and Julian Weghuber[1,2]

[1] *Center of Excellence Food Technology and Nutrition, University of Applied Sciences Upper Austria, Stelzhamerstraße 23, Wels 4600, Austria*

[2] *FFoQSI GmbH-Austrian Competence Centre for Feed and Food Quality, Safety and Innovation, Technopark 1D, Tulln 3430, Austria*

[3] *TriPlant AG, Industriestrasse 17, Buetzberg 4922, Switzerland*

* *These authors contributed equally*

Correspondence

Julian Weghuber, Center of Excellence Food Technology and Nutrition, University of Applied Sciences Upper Austria, Stelzhamerstraße 23, Wels 4600, Austria

E-mail: julian.weghuber@fh-wels.at

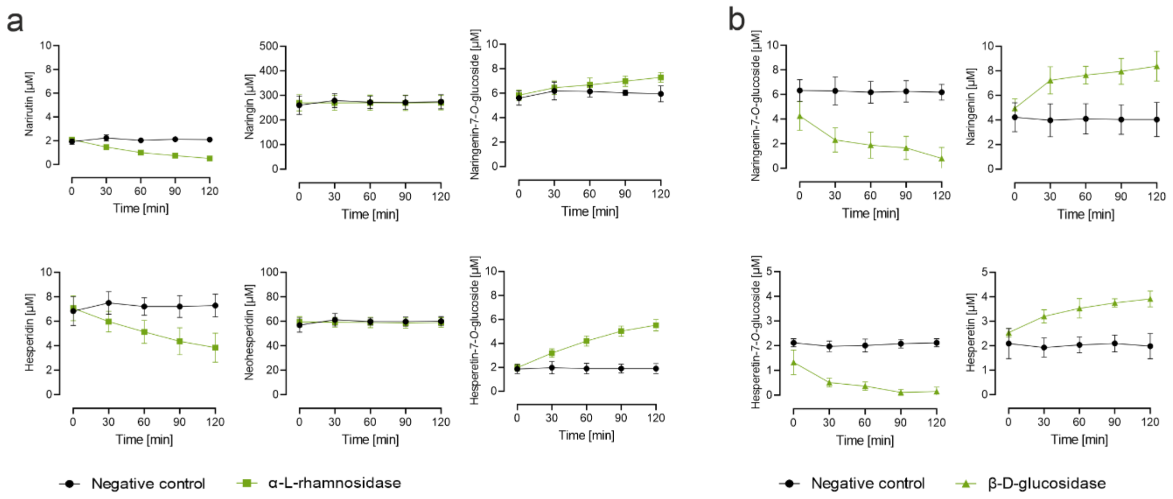

Supplementary Figure 1. Concentration of glycosylated flavanones hydrolyzed to aglycones by enzymatic treatment. a Changes in the flavanones narirutin, naringin, naringenin-7-*O*-glucoside, hesperidin, neohesperidin and hesperetin-7-*O*-glucoside during incubation of AQE with commercial α-L-rhamnosidase at 37 °C for 2 h. **b** Changes in flavanones naringenin, naringenin-7-*O*-glucoside, hesperetin, and hesperetin-7-*O*-glucoside during incubation of aqueous citrus extract AQE with commercial β-D-glucosidase at 37 °C for 2 h. No enzymes were added for negative control. Data are mean ± SD of n = 6 samples/treatment.

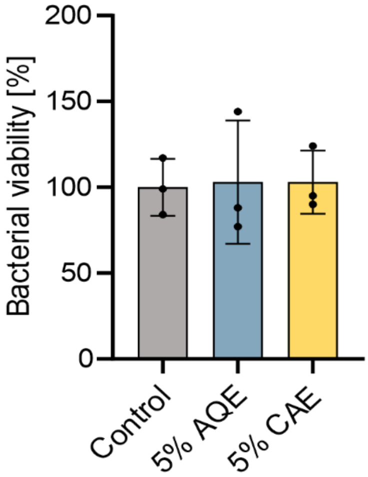

Supplementary Figure 2. Citrus extracts do not affect bacterial growth compared to control. Bacterial viability after 24 h of incubation of *Lactiplantibacillus plantarum* with 5% of aqueous extract (AQE) or citric acid hydrolyzed extract (CAE) diluted in modified MRS medium without glucose, normalized to control. Control refers to the sample that was incubated in modified MRS medium without glucose. Data are mean ± SD of n = 3 samples/treatment.

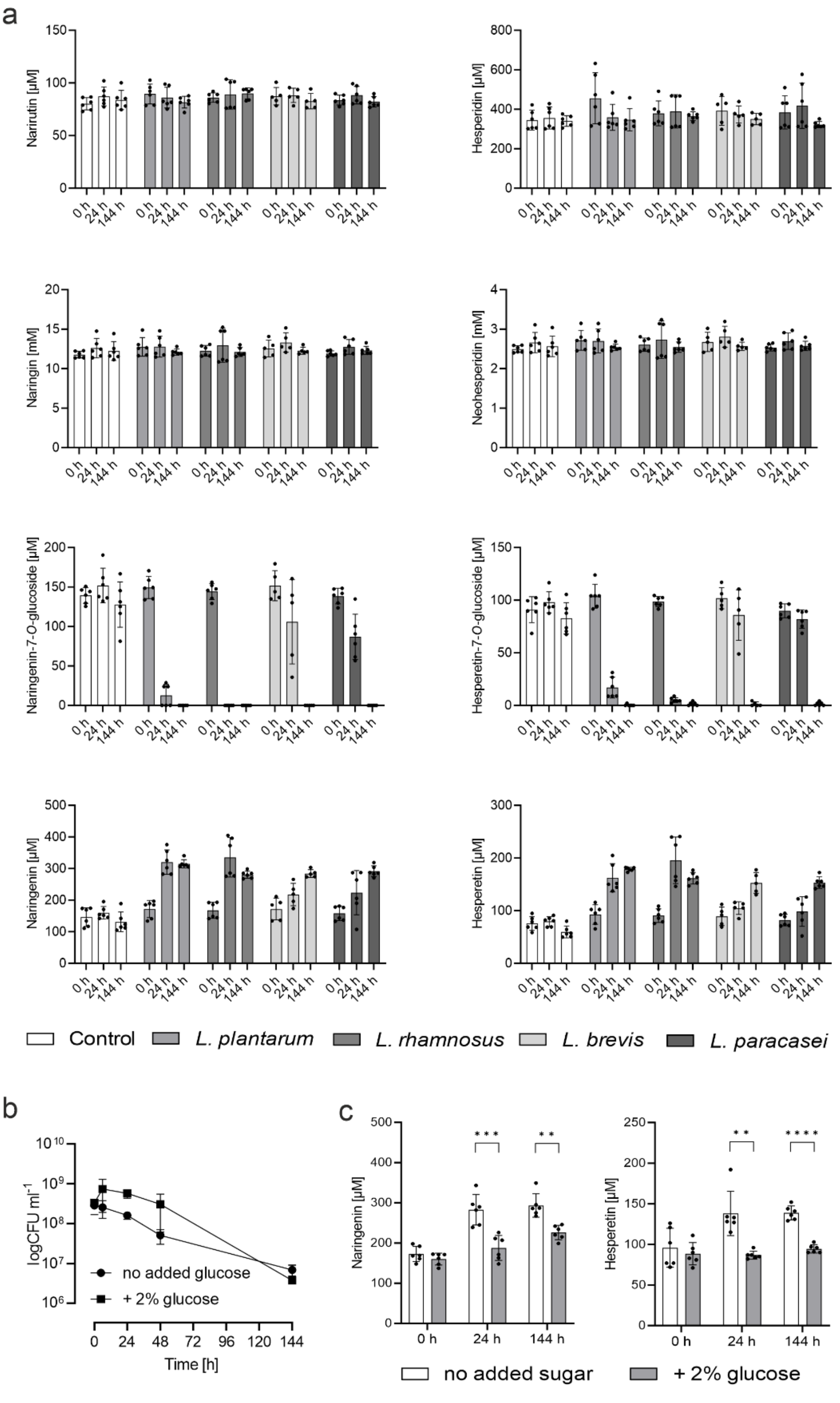

Supplementary Figure 3. Lactic acid bacteria strains metabolize 7-*O*-glucosides to aglycones in aqueous citrus extract. a Concentrations of flavanones in aqueous citrus extract (AQE) at 0 h, 24 h and 144 h of incubation with *L. plantarum*, *L. rhamnosus*, *L. brevis* or *L. paracasei* at 37 °C. Control refers to the sample without bacteria. **b** Colony formation of *L. plantarum* in AQE in presence of 2% of glucose. **c** Aglycone conversion by *L. plantarum* in presence of glucose after 24. Data are mean ± SD of n = 6 samples/treatment. Differences between treatment with and without glucose are analyzed by two-tailed unpaired t-test with Welch's correction.

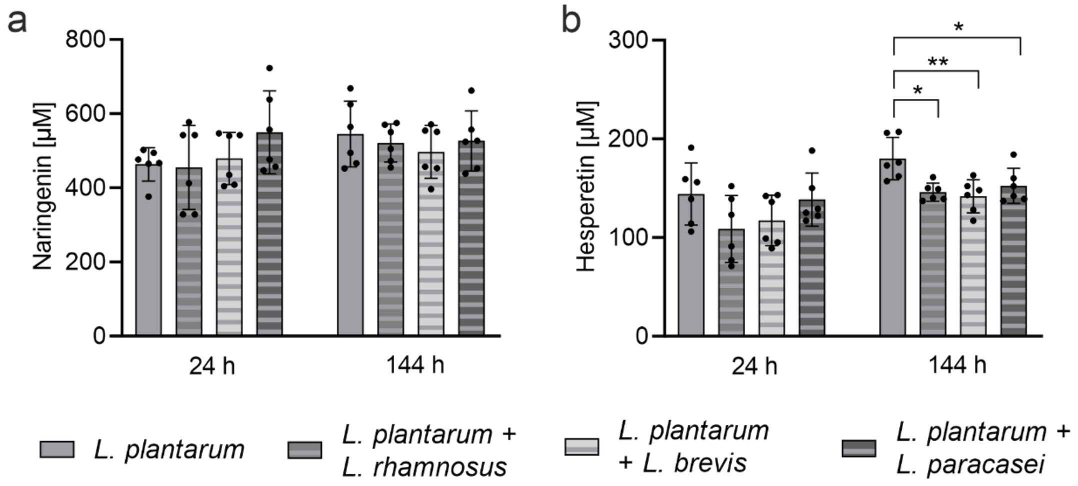

Supplementary Figure 4. Co-cultivation with different lactic acid bacteria strains does not improve aglycone concentration. Increase in concentrations of naringenin **(a)** and hesperetin **(b)** after incubation of citric acid hydrolyzed extract (CAE) with *L. plantarum* as single strain or with co-cultures of *L. plantarum* and each other strain (*L. hamnosus*, *L. brevis* or *L. paracasei*) for 24 h and 144 h. For calculation of the aglycone increase, the initial aglycone concentration (at 0 h) was subtracted from the total concentration at 24 h and 144 h, respectively. Data are mean ± SD of *n* = 6 samples/treatment. Differences between treatments are analyzed by ordinary one-way ANOVAs with Tukey's multiple comparison test.

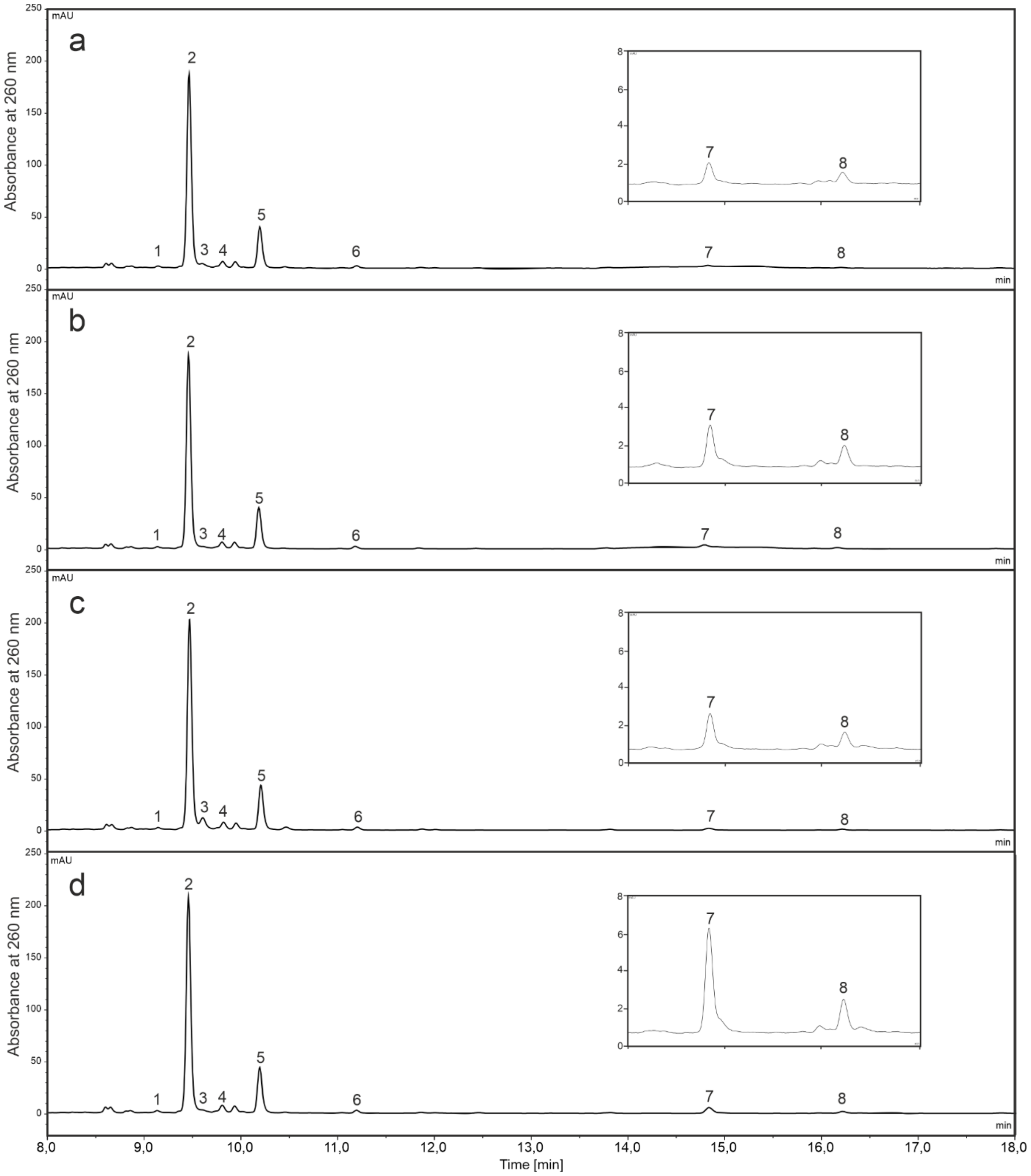

Supplementary Figure 5. Representative chromatograms of various citrus extracts under study. Chromatograms of **(a)** aqueous citrus extract (AQE), **(b)** fermented aqueous citrus extract (FermAQE), **(c)** citric acid hydrolyzed aqueous citrus extract (CAE) and **(d)** fermented citric acid hydrolyzed aqueous citrus extract (FermCAE) obtained with reversed phase chromatography with UV detection at 260 nm. Peaks represent compounds: 1 — narirutin; 2 — naringin, 3 — naringenin-7-*O*-glucoside; 4 — hesperidin; 5 — neohesperidin; 6 — hesperetin-7-*O*-glucoside; 7 — naringenin; 8 — hesperetin. The aglycones naringenin and hesperetin are shown enlarged in the separate windows, respectively.

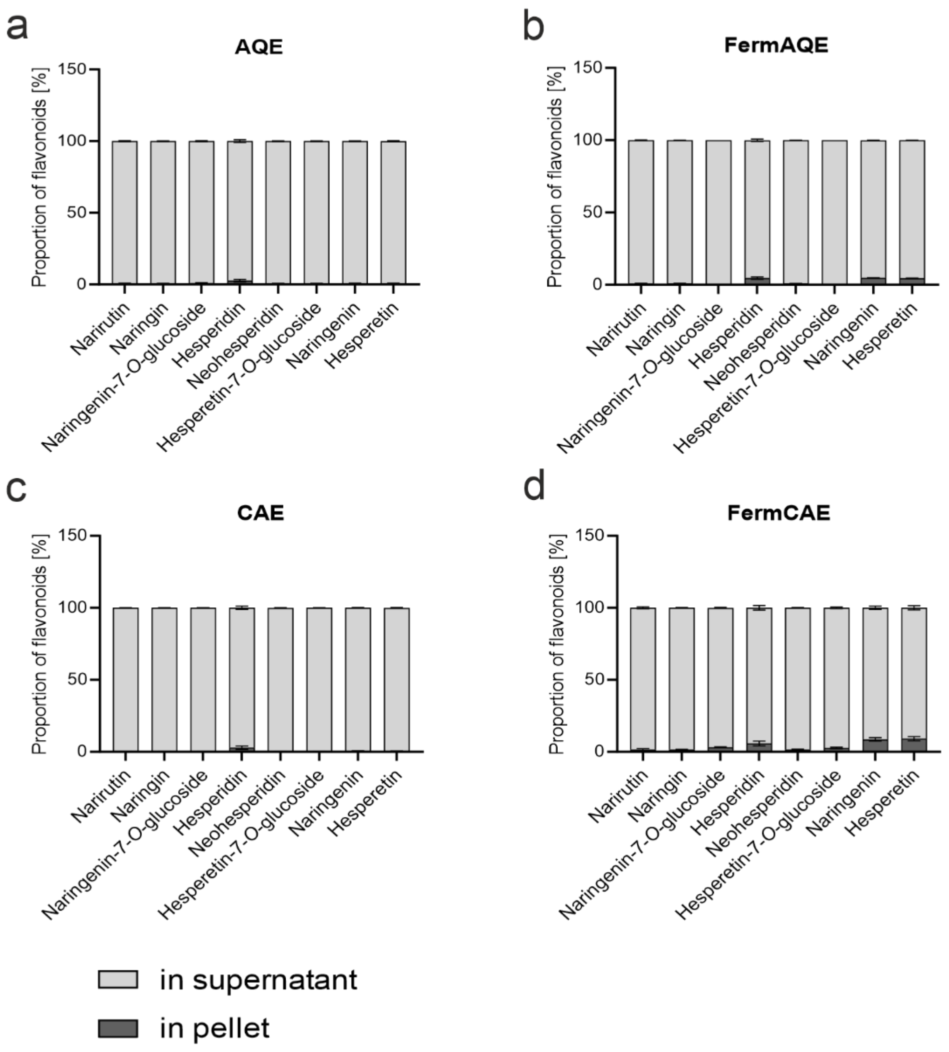

Supplementary Figure 6. Percentage of flavonoids in supernatant and pellet. The entire fermentation product includes dissolved flavonoids in the supernatant (= extract used for *in vitro* and *in vivo* experiments) and undissolved flavonoids in the pellet. Proportion of flavonoids in supernatant and pellet of **(a)** aqueous extract (AQE), **(b)** fermented aqueous extract (FermAQE), **(c)** citric acid hydrolyzed extract (CAE), and **(d)** fermented citric acid hydrolyzed extract (FermCAE). Data are mean ± SD of *n* = 3 samples/extract.

Supplementary Table 1. Influence of successive treatment with test substances and stressors on cell viability of different intestinal epithelial cells. AAPH (2,2'-azobis(2-amidinopropane) dihydrochloride) was used as stressor for Caco-2 cells and tBHP (tert-butylhydroperoxid) for IPEC-J2 cells. AQE stands for aqueous citrus extract, CAE for citric acid hydrolyzed aqueous citrus extract, FermAQE for fermented aqueous citrus extract, FermCAE for fermented citric acid hydrolyzed aqueous citrus extract. Data normalized to untreated control are mean and SD of *n* = 9 samples/treatment. Differences to control are analyzed by ordinary one-way ANOVAs with Dunnett's multiple comparison test.

Cell line	Treatment	Cell viability [% of control]		*p* value
		Mean	SD	
Caco-2	AAPH	104.80	7.29	0.2652
Caco-2	AAPH + 20 µM quercetin	104.56	5.19	0.3291
Caco-2	AAPH + 1.25% AQE	101.56	6.73	0.9993
Caco-2	AAPH + 2.5% AQE	107.74	6.90	0.0488
Caco-2	AAPH + 1.25% FermAQE	102.06	6.72	0.9959
Caco-2	AAPH + 2.5% FermAQE	105.63	7.58	0.3164
Caco-2	AAPH + 1.25% CAE	103.82	6.22	0.8143
Caco-2	AAPH + 2.5% CAE	101.46	10.18	0.9993
Caco-2	AAPH + 1.25% FermCAE	106.39	6.18	0.1751
Caco-2	AAPH + 2.5% FermCAE	106.91	7.62	0.1110
Caco-2	AAPH + 1.25% Ferm Blank	101.86	4.33	0.9990
Caco-2	AAPH + 2.5% Ferm Blank	103.48	4.69	0.8884
Caco-2	AAPH + 1.25% CA Blank	104.97	6.09	0.4878
Caco-2	AAPH + 2.5% CA Blank	103.91	7.02	0.7907
Caco-2	AAPH + 150 µM naringin	102.97	4.59	0.9514
Caco-2	AAPH + 150 µM naringenin	105.46	3.85	0.9954
Caco-2	AAPH + 150 µM hesperidin	100.00	6.45	0.1715
Caco-2	AAPH + 150 µM hesperetin	103.85	4.72	0.9995
IPEC-J2	tBHP	101.65	6.62	0.945
IPEC-J2	tBHP + 20 µM quercetin	100.05	4.57	>0.9999
IPEC-J2	tBHP + 1.25% AQE	94.97	3.60	0.0426
IPEC-J2	tBHP + 2.5% AQE	99.35	3.28	0.9996
IPEC-J2	tBHP + 1.25% FermAQE	95.43	3.23	0.0889
IPEC-J2	tBHP + 2.5% FermAQE	99.24	3.63	0.9994
IPEC-J2	tBHP + 1.25% CAE	93.90	2.30	0.0159
IPEC-J2	tBHP + 2.5% CAE	95.56	2.67	0.1821
IPEC-J2	tBHP + 1.25% FermCAE	92.94	4.05	0.0028
IPEC-J2	tBHP + 2.5% FermCAE	95.54	3.27	0.1059
IPEC-J2	tBHP + 1.25% FermBlank	98.28	3.27	0.9919
IPEC-J2	tBHP + 2.5% FermBlank	99.81	4.14	0.9999
IPEC-J2	tBHP + 1.25% CA Blank	102.52	3.77	0.9076
IPEC-J2	tBHP + 2.5% CA Blank	94.60	3.77	0.0493
IPEC-J2	tBHP + 20 µM quercetin	101.52	3.80	0.9472
IPEC-J2	tBHP + 150 µM naringin	100.72	4.46	0.9981
IPEC-J2	tBHP + 150 µM naringenin	102.10	4.21	0.8134
IPEC-J2	tBHP + 150 µM hesperidin	100.26	4.08	0.9998

Supplementary Movie. Impact of citrus extracts of different biotransformation level on cell migration of IPEC-J2 cells under challenge conditions. Representative video file of cell migration of IPEC-J2 cells pre-treated with citrus extracts for 6 h and stressed with tert-butylhydroperoxide (tBHP) after scratching. Cell migration is shown between 15 to 420 min.

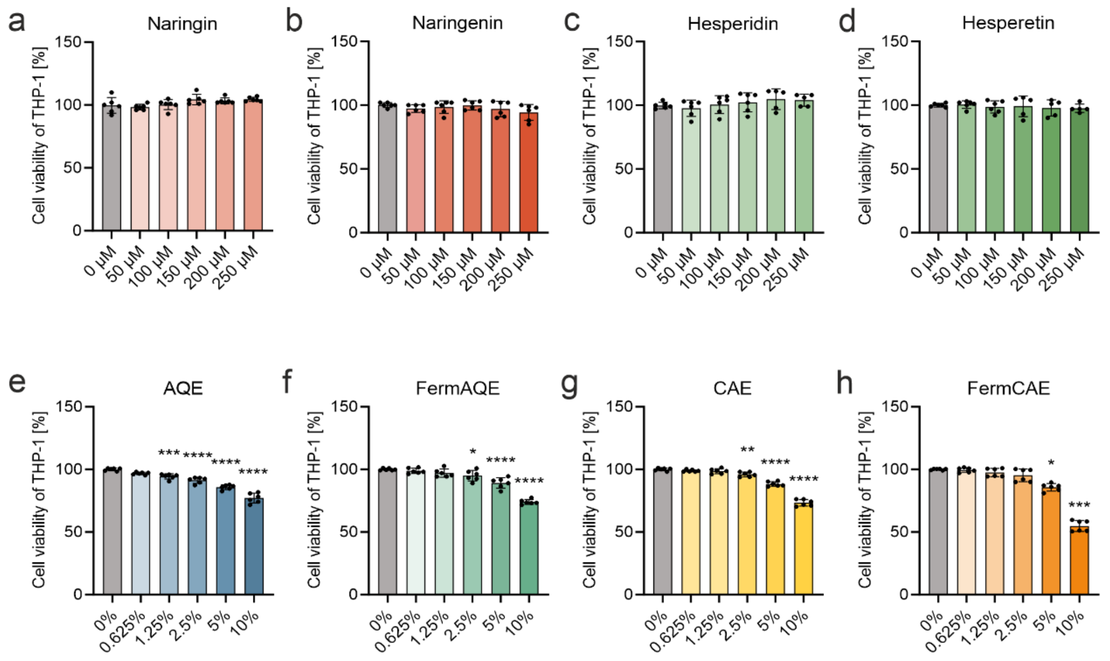

Supplementary Figure 7. Influence of flavanones and citrus extracts on cell viability of human differentiated THP-1 cells. Cell viability of human THP-1 cells treated with increasing concentrations of naringin **(a)**, naringenin **(b)**, hesperidin **(c)**, hesperetin **(d)**, AQE **(e)** FermAQE **(f)**, CAE **(g)** or FermCAE **(h)**, normalized to untreated control. AQE stands for aqueous citrus extract, CAE for citric acid hydrolyzed aqueous citrus extract, FermAQE for fermented aqueous citrus extract, FermCAE for fermented citric acid hydrolyzed aqueous citrus extract. Data are mean ± SD of n = 6 samples/treatment. Differences to control are analyzed by ordinary one-way ANOVAs with Dunnett's multiple comparison test. Substance concentrations with a resulting cell viability of >90% are accepted for future experiments.

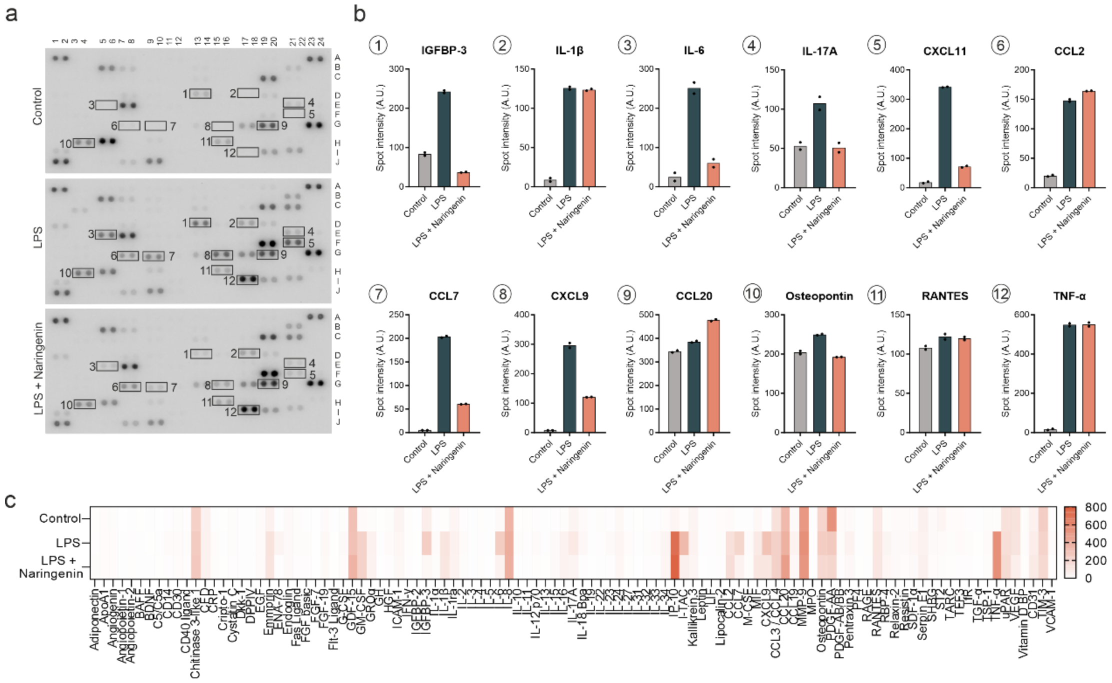

Supplementary Figure 8. Cytokine expression profiles of LPS-stimulated THP-1 cells treated with naringenin. a Representative images of cytokine arrays incubated with supernatants of differentiated THP-1 macrophages treated with 150 µM of naringenin under lipopolysaccharide (LPS) challenge. Expressed cytokines are presented as duplicate spots. Relevant cytokines are outlined and marked with numbers. The corresponding spot intensities (stated as arbitrary units, A.U.) of these analytes are presented in **(b)**. Data of technical duplicates are shown. **c** Heat map of 105 cytokines presented as mean spot intensities in a color range from white (low level) to red (high level).

Supplementary Table 2. Information on calibration curve range, calibration type, coefficient of determination (R^2), limit of detection (LOD; S:N = 3:1), and limit of quantification (LOQ; S:N = 10:1).

Substance	Calibration curve range [µM]	Calibration type	R^2	LOD [µM]	LOQ [µM]
Narirutin	0–1481.38	Linear	0.9971	0.0093	0.0311
Naringin	0–791.44	Linear	1.0000	0.0088	0.0293
Naringenin-7-*O*-glucoside	0–2256.04	Linear	0.9999	0.0103	0.0342
Hesperidin	0–321.02	Linear	0.9998	0.0110	0.0368
Neohesperidin	0–1899.90	Linear	1.0000	0.0128	0.0425
Hesperetin-7-*O*-glucoside	0–1722.65	Linear	0.9999	0.0185	0.0616
Naringenin	0–4040.26	Linear	0.9999	0.0531	0.1770
Hesperetin	0–4035.99	Linear	0.9998	0.0355	0.1182

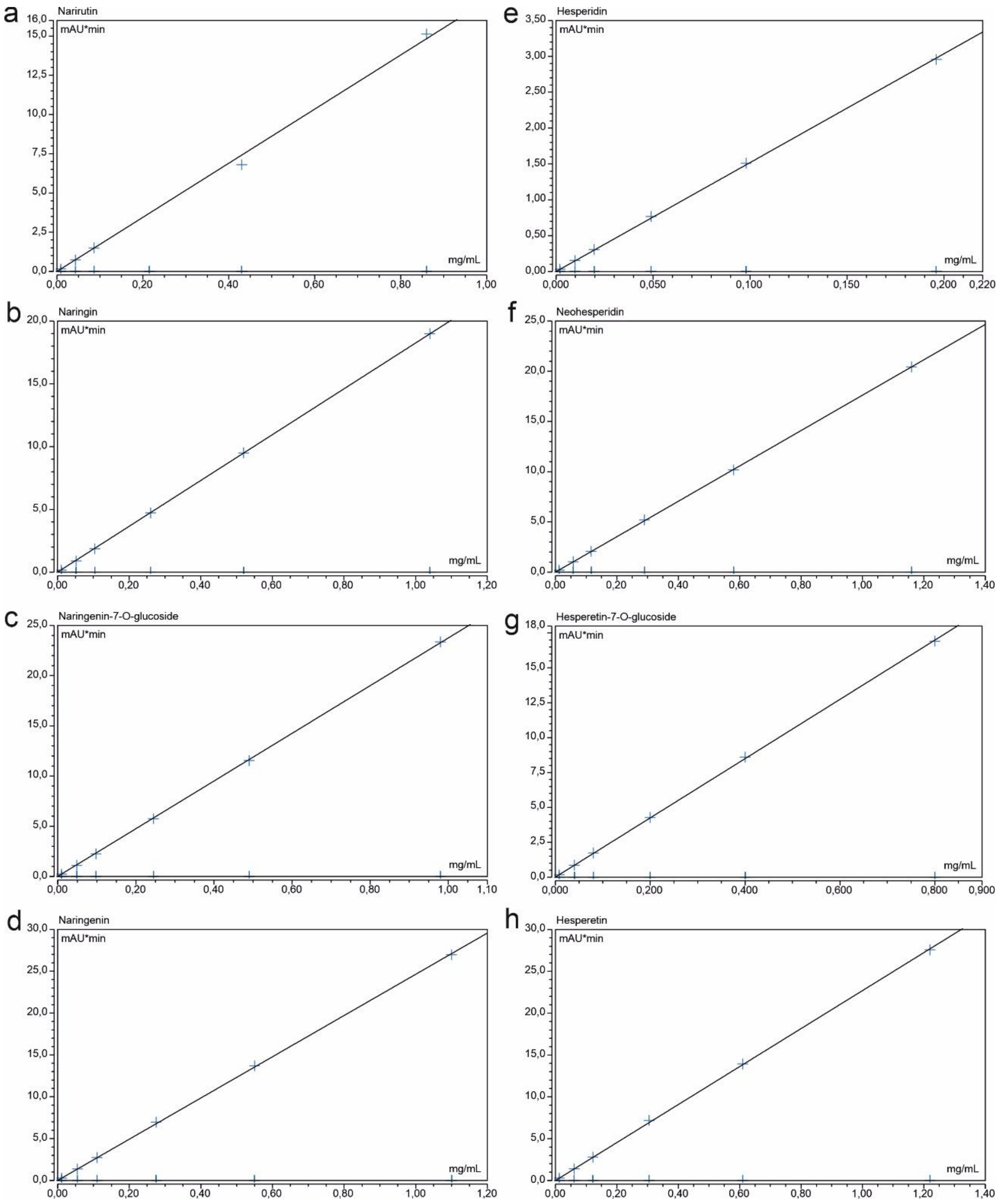

Supplementary Figure 9. Calibration curves of the standards used for HPLC analysis. Calibration curves for narirutin **(a)**, naringin **(b)**, naringenin-7-*O*-glucoside **(c)**, naringenin **(d)**, hesperidin **(e)**, neohesperidin **(f)**, hesperetin-7-*O*-glucoside **(g)**, hesperetin **(h)**.

Supplementary Table 3. Performance parameters of multiplex cytokine assay. Target analytes are CC-chemokine ligand 7 (CCL7), C-X-C motif chemokine 11 (CXCL11), C-X-C motif chemokine 9 (CXCL9), interleukin 6 (IL-6), and tumor necrosis factor α (TNF-α).

Performance parameters	Assay number	CCL7	CXCL11	CXCL9	IL-6	TNFα	Inter- and intra-assay mean
Calibration goodness-of-fit R^2	1	0.989	0.987	0.993	0.987	0.989	0.989
	2	0.997	0.998	0.997	0.997	0.996	0.997
	3	0.999	0.997	0.999	0.999	0.997	0.998
	Mean	0.995	0.994	0.997	0.994	0.994	0.995
Coefficient of variation CV [%]	1	11.123	8.540	4.194	4.397	3.675	6.386
	2	8.330	5.259	1.472	3.196	3.822	4.416
	3	2.392	15.168	0.293	3.306	3.771	4.986
	Mean	7.282	9.656	1.986	3.633	3.756	5.263
Standards recovery [%]	1	100.271	100.399	101.288	101.848	101.844	101.130
	2	100.363	100.213	100.470	100.306	100.558	100.382
	3	99.397	100.337	100.070	100.228	100.404	100.087
	Mean	100.011	100.316	100.609	100.794	100.936	100.533

Supplementary Table 4. Parameters of statistical evaluation with ordinary one-way ANOVA test for the data sets that passed Shapiro-Wilk test for normality of data distribution.

Graph	Multiple comparison test	Number of experiments	*n* per treatment group of all experiments	Technical replicates	ANOVA summary		
					F	DFn	DFd
Fig. 2b. Hesperetin	Dunnett	2	6	not applicable (n. a.)	57.85	4	25
Fig. 3b. Narirutin	Dunnett	4	4	n. a.	1.54	3	12
Fig. 3b. Naringin	Dunnett	4	4	n. a.	10.91	3	12
Fig. 3b. Naringenin-7-*O*-glucoside	Dunnett	4	4	n. a.	80.64	3	12
Fig. 3b. Neohesperidin	Dunnett	4	4	n. a.	9.78	3	12
Fig. 3b. Hesperetin-7-*O*-glucoside	Dunnett	4	4	n. a.	291.00	3	12
Fig. 3d. Narirutin	Tukey	3	9	n. a.	6.52	3	32
Fig. 3d. Neohesperidin	Tukey	3	9	n. a.	2.73	3	32
Fig. 3d. Hesperetin	Tukey	3	9	n. a.	1.20	3	32
Fig. 4b. Uptake, Comparison	Šídák	2	6	n.a.	218.10	3	20
Fig. 4c. Uptake, AQE	Šídák	2	6	n.a.	59.72	3	20
Fig. 4d. Uptake, FermCAE	Šídák	2	6	n.a.	412.30	3	20
Fig. 4f. Transport rate, AQE	Šídák	2	6	n.a.	454.90	4	25
Fig. 4g. Transport rate, FermCAE	Šídák	2	6	n.a.	498.70	4	25
Fig. 4h. Transport, Comparison	Šídák	2	6	n.a.	478.10	3	20
Fig. 5a. ROS level, Caco-2, flavanones	Šídák	3	9	n.a.	566.70	6	56
Fig. 5b. ROS level, Caco-2, extracts	Šídák	3	9	n.a.	383.80	14	147
Fig. 5c. ROS level, IPEC-J2, flavanones	Šídák	3	9	n.a.	154.60	6	56
Fig. 5d. ROS level, IPEC-J2, extracts	Šídák	3	9	n.a.	123.00	14	141
Fig. 5e. Cell front velocity	Šídák	3	6	n.a.	61.83	5	30
Fig. 7b. IL-6	Šídák	2	4	2	69.92	5	18
Fig. 7c. CXCL11	Šídák	2	4	2	13.21	5	18

Graph	Multiple comparison test	Number of experiments	*n* per treatment group of all experiments	Technical replicates	ANOVA summary		
					F	DFn	DFd
Fig. 7d. CCL7	Šídák	2	4	2	68.16	5	17
Fig. 7f. TNF-α	Šídák	2	4	2	12.66	5	18
Fig. 7g. IL-6	Šídák	2	4	2	103.10	10	33
Fig. 7h. CXCL11	Šídák	2	4	2	13.09	10	33
Fig. 7i. CCL7	Šídák	2	4	2	15.68	10	33
Fig. 7j. CXCL9	Šídák	2	4	2	9.18	10	32
Fig. 7k. TNF-α	Šídák	2	4	2	19.45	10	33
Fig. 8c. Mortality, intestinal challenge	Šídák	3	12	n. a.	333.00	5	66
Fig. 8d. Smurf phenotype, intestinal challenge	Šídák	3	12	n. a.	41.98	5	66
Fig. 8e. ROS level, oxidative stress	Šídák	3	12	2	24.59	5	66
Fig. 8f. Metabolic rate, oxidative stress	Šídák	3	12	2	15.41	5	66
Fig. 8g. Mortality, oxidative stress	Šídák	3	9	n. a.	22.09	5	48
Fig. 8h. Climbing performance, oxidative stress	Šídák	3	9	n. a.	69.00	5	48
Supp. Fig. 3a. Naringenin, t24	Tukey	2	6	n. a.	1.38	3	20
Supp. Fig. 3a. Naringenin, t144	Tukey	2	6	n. a.	0.422	3	20
Supp. Fig. 3b. Hesperetin, t24	Tukey	2	6	n. a.	1.94	3	20
Supp. Fig. 3b. Hesperetin, t144	Tukey	2	6	n. a.	6.26	3	20
Supp. Table 1. Cell viability, IPEC-J2, flavanones	Dunnett	3	9	n. a.	1.01	6	56
Supp. Table 1. Cell viability, IPEC-J2, extracts	Dunnett	3	9	n. a.	4.63	12	120
Supp. Table 1. Cell viability, Caco-2, flavanones	Dunnett	3	9	n. a.	2.07	6	56
Supp. Table 1. Cell viability, Caco-2, extracts	Dunnett	3	9	n. a.	1.30	14	147
Supp. Fig. 6a. Cell viability, THP-1, Naringin	Dunnett	2	6	n. a.	2.96	5	30
Supp. Fig. 6b. Cell viability, THP-1, Naringenin	Dunnett	2	6	n. a.	1.32	5	30
Supp. Fig. 6c. Cell viability, THP-1, Hesperidin	Dunnett	2	6	n. a.	1.01	5	28
Supp. Fig. 6d. Cell viability, THP-1, Hesperetin	Dunnett	2	6	n. a.	0.32	5	27
Supp. Fig. 6e. Cell viability, THP-1, AQE	Dunnett	2	6	n. a.	80.07	5	30
Supp. Fig. 6f. Cell viability, THP-1, FermAQE	Dunnett	2	6	n. a.	61.57	5	30
Supp. Fig. 6g. Cell viability, THP-1, CAE	Dunnett	2	6	n. a.	194.00	5	30

Supplementary Table 5. Parameters of statistical evaluation with Kruskal-Wallis test with Dunn's multiple comparison test for the data sets that did not pass Shapiro-Wilk test for normality of data distribution.

Graph	Number of experiments	*n* per treatment group of all experiments	Technical replicates	Kruskal-Wallis summary		
				H	DFn	DFd
Fig. 2b. Narirutin	2	6	n. a.	4.13	4	25
Fig. 2b. Naringin	2	6	n. a.	4.23	4	25
Fig. 2b. Naringenin-7-*O*-glucoside	2	6	n. a.	20.38	4	25
Fig. 2b. Naringenin	2	6	n. a.	26.50	4	25
Fig. 2b. Hesperidin	2	6	n. a.	5.38	4	25
Fig. 2b. Neohesperidin	2	6	n. a.	3.86	4	25
Fig. 2b. Hesperetin-7-*O*-glucoside	2	6	n. a.	17.51	4	25
Fig. 3b. Hesperidin	2	4	n. a.	1.96	3	12
Fig. 3c. Naringenin	2	5	n. a.	16.09	4	16
Fig. 3c. Hesperetin	2	5	n. a.	14.66	4	16
Fig. 3d. Naringin	3	9	n. a.	14.78	3	32
Fig. 3d. Naringenin-7-*O*-glucoside	3	9	n. a.	30.85	3	32
Fig. 3d. Naringenin	3	9	n. a.	32.84	3	32
Fig. 3d. Hesperidin	3	9	n. a.	2.23	3	31
Fig. 3d. Hesperetin-7-*O*-glucoside	3	9	n. a.	29.85	3	32
Fig. 6b. Cytokines 1–12	1	1	2	6.67	3	4
Fig. 8e. CXCL9	3	4	2	15.02	5	18
Supp. Fig. 6h. Cell viability, THP-1, FermCAE	2	6	n. a.	26.11	5	30
Supp. Fig. 7b. Cytokines 1–12	1	1	2	4.57	3	4

Supplementary Table 6. Parameters of statistical evaluation with unpaired two-tailed t-test with Welch's correction for the data sets that passed Shapiro-Wilk test for normality of data distribution.

Graph	Number of experiments	*n* per treatment group of all experiments	Technical replicates	t-test summary		
				t	DFn	DFd
Supp. Fig. 3c. Naringenin, t0	2	6	n. a.	1.29	5	5
Supp. Fig. 3c. Naringenin, t24	2	6	n. a.	4.73	5	5
Supp. Fig. 3c. Naringenin, t144	2	6	n. a.	4.82	5	5
Supp. Fig. 3c. Hesperetin, t0	2	6	n. a.	0.65	5	5
Supp. Fig. 3c. Hesperetin, t24	2	6	n. a.	4.55	5	5
Supp. Fig. 3c. Hesperetin, t144	2	6	n. a.	10.81	5	5
