# Supplementary material for: Combined acid hydrolysis and fermentation improves bioactivity of citrus flavonoids in vitro and in vivo
Source: Commun Biol. 2023 Oct 25;6:1083. doi: 10.1038/s42003-023-05424-7 (PMC10600125; doi:10.1038/s42003-023-05424-7)
Supplement: Supplementary file 2 — Description of Supplementary Materials [file 42003_2023_5424_MOESM2_ESM.docx]

**Description of Additional Supplementary Files**

**File name:** Supplementary Data

**Description:** The source data behind the graphs in the paper.

**File name:** Supplementary Movie

**Description:** Impact of citrus extracts of different biotransformation level on cell migration of IPEC-J2 cells under challenge conditions. Representative video file of cell migration of IPEC-J2 cells pre-treated with citrus extracts for 6 h and stressed with tertbutylhydroperoxide (tBHP) after scratching. Cell migration is shown between 15 to 420 min.
