# Supplementary material for: Combined acid hydrolysis and fermentation improves bioactivity of citrus flavonoids in vitro and in vivo
Source: Commun Biol. 2023 Oct 25;6:1083. doi: 10.1038/s42003-023-05424-7 (PMC10600125; doi:10.1038/s42003-023-05424-7)
Supplement: Supplementary file 5 — Reporting summary [file 42003_2023_5424_MOESM5_ESM.pdf]

nature portfolio

Corresponding author(s): Julian Weghuber

Last updated by author(s): Sep 25, 2023

Reporting Summary

Nature Portfolio wishes to improve the reproducibility of the work that we publish. This form provides structure for consistency and transparency in reporting. For further information on Nature Portfolio policies, see our Editorial Policies and the Editorial Policy Checklist.

Statistics

For all statistical analyses, confirm that the following items are present in the figure legend, table legend, main text, or Methods section.

n/a	Confirmed	
☐	☒	The exact sample size (*n*) for each experimental group/condition, given as a discrete number and unit of measurement
☐	☒	A statement on whether measurements were taken from distinct samples or whether the same sample was measured repeatedly
☐	☒	The statistical test(s) used AND whether they are one- or two-sided<br>*Only common tests should be described solely by name; describe more complex techniques in the Methods section.*
☐	☒	A description of all covariates tested
☐	☒	A description of any assumptions or corrections, such as tests of normality and adjustment for multiple comparisons
☐	☒	A full description of the statistical parameters including central tendency (e.g. means) or other basic estimates (e.g. regression coefficient) AND variation (e.g. standard deviation) or associated estimates of uncertainty (e.g. confidence intervals)
☐	☒	For null hypothesis testing, the test statistic (e.g. *F*, *t*, *r*) with confidence intervals, effect sizes, degrees of freedom and *P* value noted<br>*Give P values as exact values whenever suitable.*
☒	☐	For Bayesian analysis, information on the choice of priors and Markov chain Monte Carlo settings
☒	☐	For hierarchical and complex designs, identification of the appropriate level for tests and full reporting of outcomes
☒	☐	Estimates of effect sizes (e.g. Cohen's *d*, Pearson's *r*), indicating how they were calculated

Our web collection on statistics for biologists contains articles on many of the points above.

Software and code

Policy information about availability of computer code

Data collection	HPLC data was collected using the Chromeleon™ software (Version 7.3.1, Thermo Scientific™ Dionex™, Waltham, MA, USA).<br>All fluorescence and absorbance measurements were processed with Omega Software, Version 2.10 and Mars Data Analysis Software, Version 2.30 (both BMG LABTECH GmbH).<br>For the cytokine multiplex immunoassay, data was acquired by Luminex® 200™ (Luminex Corp., Austin, TX, USA).
Data analysis	Statistical tests were performed using GraphPad Prism (Version 9.4.1 GraphPad Software, SanDiego, CA, USA).<br>For the cytokine array, semi-quantitative comparison of data was performed using the Image Lab Software (Bio-Rad Laboratories).<br>For the cytokine multiplex immunoassay, data was analyzed with xPonent® acquisition software, Version 4.3 (Luminex Corp., Austin, TX, USA).

For manuscripts utilizing custom algorithms or software that are central to the research but not yet described in published literature, software must be made available to editors and reviewers. We strongly encourage code deposition in a community repository (e.g. GitHub). See the Nature Portfolio guidelines for submitting code & software for further information.

Data

Policy information about availability of data

All manuscripts must include a data availability statement. This statement should provide the following information, where applicable:

- Accession codes, unique identifiers, or web links for publicly available datasets
- A description of any restrictions on data availability
- For clinical datasets or third party data, please ensure that the statement adheres to our policy

Numerical source data for all charts and graphs can be found in Supplementary Data. Further data that support the findings of this study are not openly available due to reasons of sensitivity and are available from the corresponding author upon reasonable request.

Research involving human participants, their data, or biological material

Policy information about studies with human participants or human data. See also policy information about sex, gender (identity/presentation), and sexual orientation and race, ethnicity and racism.

Reporting on sex and gender	not relevant
Reporting on race, ethnicity, or other socially relevant groupings	not relevant
Population characteristics	not relevant
Recruitment	not relevant
Ethics oversight	not relevant

Note that full information on the approval of the study protocol must also be provided in the manuscript.

Field-specific reporting

Please select the one below that is the best fit for your research. If you are not sure, read the appropriate sections before making your selection.

☒ Life sciences ☐ Behavioural & social sciences ☐ Ecological, evolutionary & environmental sciences

For a reference copy of the document with all sections, see nature.com/documents/nr-reporting-summary-flat.pdf

Life sciences study design

All studies must disclose on these points even when the disclosure is negative.

Sample size	For Drosophila studies groups sizes commonly applied in this field of research were used. Sufficiency of living organisms number was controlled with resource equation approach and acceded calculated minimal values.
Data exclusions	Outliers were excluded from the data based on the results of the ROUT outlier test (GraphPad Software).
Replication	Experiments were repeated and could be verified.
Randomization	Randomization was performed manually: cell culture samples (where relevant) or groups with age-synchronized Drosophila melanogaster were randomly assigned to the treatment groups, while originating from the same batch and being processed at the same time points in standardized conditions.
Blinding	Blinding was not relevant to the study because no humans were included.

Reporting for specific materials, systems and methods

We require information from authors about some types of materials, experimental systems and methods used in many studies. Here, indicate whether each material, system or method listed is relevant to your study. If you are not sure if a list item applies to your research, read the appropriate section before selecting a response.

Materials & experimental systems

n/a	Involved in the study	
☐	☒	Antibodies
☐	☒	Eukaryotic cell lines
☒	☐	Palaeontology and archaeology
☐	☒	Animals and other organisms
☒	☐	Clinical data
☒	☐	Dual use research of concern
☒	☐	Plants

Methods

n/a	Involved in the study	
☒	☐	ChIP-seq
☒	☐	Flow cytometry
☒	☐	MRI-based neuroimaging

Antibodies

Antibodies used	The Proteome Profiler™ Array and the Luminex xMAP® custom multiplex assay (both Bio-Techne Ltd., UK) included antibody cocktails.
Validation	Validation statement on the manufacturer's website: In addition to our established validation procedures, we also implement the 5 Pillars of Antibody Validation: Genetic Strategy Validation, Orthogonal Validation, Independent Antibody Validation, Expression of Tagged Proteins Validation and Biological Strategies Validation.

Eukaryotic cell lines

Policy information about cell lines and Sex and Gender in Research

Cell line source(s)	German Collection of Microorganisms and Cell Cultures GmbH; IPEC-J2 (ACC 701) and human Caco-2 (ACC 169); THP-1 (TIB-202™) monocytes were obtained from American Type Culture Collection
Authentication	Cell lines were obtained from commercial providers but not further authenticated
Mycoplasma contamination	used cell lines were tested negative
Commonly misidentified lines (See ICLAC register)	none

Animals and other research organisms

Policy information about studies involving animals; ARRIVE guidelines recommended for reporting animal research, and Sex and Gender in Research

Laboratory animals	Drosophila melanogaster, strain w1118, female 1-14 d.o.
Wild animals	Study did not involve wild animals
Reporting on sex	Female Drosophila melanogaster was applied in the studies and reported results are observed in female fruit fly. Choice of sex was related to technical side of the experiments, e. g. identification of Smurfs is commonly performed in female fruit fly due to its bigger size. However, authors of the manuscript do not state that the observed effects are exclusively sex-related, neither they state that the observed effect is true for both sexes
Field-collected samples	Study did not involve samples collected on the field
Ethics oversight	No ethical approval was required

Note that full information on the approval of the study protocol must also be provided in the manuscript.
